# Guided Heterostructure Growth of CoFe LDH on Ti_3_C_2_T_x_ MXene for Durably High Oxygen Evolution Activity

**DOI:** 10.1002/smll.202404927

**Published:** 2024-09-10

**Authors:** Jiali Sheng, Jiahui Kang, Pan Jiang, Kristoffer Meinander, Xiaodan Hong, Hua Jiang, Nonappa, Olli Ikkala, Hannu‐Pekka Komsa, Bo Peng, Zhong‐Peng Lv

**Affiliations:** ^1^ Department of Applied Physics Aalto University ESPOO FIN‐02150 Finland; ^2^ Research Institute of Wood Industry Chinese Academy of Forestry Xiangshan Road Beijing 100091 China; ^3^ Department of Bioproducts and Biosystems Aalto University Espoo FIN‐02150 Finland; ^4^ Faculty of Engineering and Natural Sciences Tampere University Tampere FI‐33101 Finland; ^5^ Microelectronics Research Unit Faculty of Information Technology and Electrical Engineering University of Oulu Oulu FIN‐90014 Finland; ^6^ Department of Materials Science Advanced Coating Research Center of Ministry of Education of China Fudan University Shanghai 200433 China

**Keywords:** guided growth, layered double hydroxides, modelling, oxygen evolution reaction, Ti_3_C_2_T_x_ MXene

## Abstract

Heterostructures of layered double hydroxides (LDHs) and MXenes have shown great promise for oxygen evolution reaction (OER) catalysts, owing to their complementary physical properties. Coupling LDHs with MXenes can potentially enhance their conductivity, stability, and OER activity. In this work, a scalable and straightforward in situ guided growth of CoFeLDH on Ti_3_C_2_T_x_ is introduced, where the surface chemistry of Ti_3_C_2_T_x_ dominates the resulting heterostructures, allowing tunable crystal domain sizes of LDHs. Combined simulation results of Monte Carlo and density functional theory (DFT) validate this guided growth mechanism. Through this way, the optimized heterostructures allow the highest OER activity of the overpotential = 301 mV and Tafel slope = 43 mV dec^−1^ at 10 mA cm^−2^, and a considerably durable stability of 0.1% decay over 200 h use, remarkably outperforming all reported LDHs‐MXenes materials. DFT calculations indicate that the charge transfer in heterostructures can decrease the rate‐limiting energy barrier for OER, facilitating OER activity. The combined experimental and theoretical efforts identify the participation role of MXene in heterostructures for OER reactions, providing insights into designing advanced heterostructures for robust OER electrocatalysis.

## Graphical abstract

Challenges remain for growth high performance and robust heterostructures of 2D layered double hydroxides (LDH) and MXenes, which hold great promise for energy conversion systems. By selecting different Ti_3_C_2_T_x_ MXene surface chemistry, the researchers discovered that the growth of CoFe LDH nanosheets are guided by the terminations on Ti_3_C_2_T_x_ surface. The resulting LDH‐MXene heterostructure shows surprisingly robust oxygen evolution reaction performance.

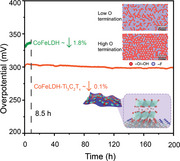

## 1 Introduction

The massive use of renewable energy sources, instead of fossil fuels, is crucial for a sustainable future.^[^ ^1^, ^2^, ^3^, ^4^ ^]^ Green hydrogen, derived from water electrolysis, offers a compelling alternative to fossil fuels, because of its high energy density and zero‐carbon footprint.^[^ ^5^ ^]^ Within the water‐splitting process, the oxygen evolution reaction (OER) at the anode poses a significant bottleneck due to its high overpotential and sluggish kinetics. This multi‐step electron‐proton transfer process involves complex oxygen intermediates.^[^ ^6^, ^7^, ^8^ ^]^ While cutting‐edge OER electrocatalysts like RuO_2_ and IrO_2_ demonstrate exceptional activity, their scarcity and cost hinder widespread implementation.^[^ ^9^, ^10^ ^]^ Therefore, developing cost‐effective, earth‐abundant OER electrocatalysts with outstanding catalytic performance is imperative for the large‐scale adoption of green hydrogen.

Layered double hydroxides (LDHs) from abundant elements such as Co, Fe, and Ni, have emerged as promising, low‐cost electrocatalysts for OER in alkaline solutions. LDHs, ultrathin 2‐dimensional (2D) nanosheets with rich chemical compositions, possess significant advantages for electrocatalysts, including high specific surface area, dense active sites from surface vacancy defects, and rich chemical compositions.^[^ ^11^, ^12^, ^13^, ^14^ ^]^ The catalytically active phase, reaction center, and the OER mechanism of LDHs have also been revealed using a combination of experiments and density functional theory (DFT).^[^ ^15^, ^16^ ^]^ Significant challenges still exist to further enhancing LDHs’ catalytic activity owing to their intrinsic poor conductivity, limited electron and charge transfer ability, and scarcity of active edge sites.^[^ ^17^ ^]^

To break the catalytic limitation of LDHs, the formation of heterostructures by incorporating LDHs with various 2D materials, such as dichalcogenides,^[^ ^18^ ^]^ graphene,^[^ ^19^, ^20^ ^]^ and MXenes^[^ ^21^, ^22^, ^23^, ^24^, ^25^, ^26^ ^]^ has been attempted. The general strategy is to introduce these (semi)conductive substrates to confine the LDH growth in their layered or mesoporous structures, and to enhance the conductivity and/or charge transfer ability, thus improving catalytic performance.^[^ ^27^ ^]^ For instance, LDH‐graphene heterostructures were reported with superior OER activity, despite the morphology of LDH was not affected.^[^ ^19^, ^20^ ^]^ Differing from other 2D materials with simple surface chemistry, MXenes possess diverse surface terminations (‐O, ‐OH, and ‐F), offering possibilities in morphological impact of LDHs. On the other hand, the heterogenization of LDHs and MXene may allow complementary merits.^[^ ^28^ ^]^ Specifically, the electronic coupling at the interface of the LDH‐MXene heterostructures improves the conductivity.^[^ ^29^, ^30^ ^]^ The charge redistribution by charge transfer between two materials can stabilize the chemical structure and enhance the electrochemical activity.^[^ ^31^, ^32^ ^]^ However, previous work has primarily focused on simple compositions and charge transfer at heterointerfaces. The role of MXene surface chemistry in the growth, structure, and catalytic performance of LDH‐MXene heterostructures remains subtle, requiring efforts both in experiment and theory.

In this work, we developed the surface chemistry‐guided growth of Co(II)Fe(III)LDH (CoFeLDH) nanosheets on Ti_3_C_2_T_x_ MXene surface for durable robust OER catalysis. Monte‐Carlo and DFT methods are employed to reveal the affinity preference of LDH precursor to the Ti_3_C_2_T_x_ surface terminations (−O, −OH, and −F). Theoretical results are consistent with experimental observations, showing that the O‐terminations on the Ti_3_C_2_T_x_ surface effectively confine the CoFeLDH domain size. This confinement leads to increased edge defects and a reduction in the CoFeLDH layer thickness. The optimized heterostructures demonstrate the highest OER activity (overpotential = 301 mV, Tafel slope = 43 mV dec⁻^1^ at 10 mA cm⁻^2^) and exceptional durability with only 0.1% decay over 200 h of catalytic use, surpassing all previously reported LDHs‐MXenes heterostructures. The structure evolution and reconstruction of the catalyst is also investigated. We further identify an electron transfer from Ti_3_C_2_T_x_ to γCoFeLDH by DFT during OER catalysis, accounting for the enhanced electrocatalytic performance. These results provide insightful mechanism and strategy in the design of advanced OER catalysts.

## 2 Results and Discussion

### 2.1 Ti_3_C_2_T_x_ MXene with Tunable Surface Chemistry

**Figure 1:**
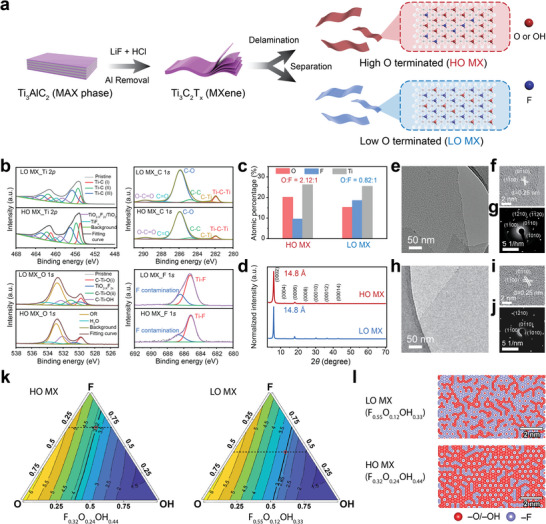
Preparation, characterization, and simulations of Ti_3_C_2_T_x_ MXene with different O to F termination ratios. a) Schematics of the synthesis and separation of HO MX and LO MX Ti_3_C_2_T_x_ MXenes. b) Comparison of Ti 2p, C 1s, O 1s, and F 1s spectra from HO MX and LO MX; c) Atomic percentage of −O/−OH, −F, and Ti in HO MX and LO MX determined by XPS; d) XRD patterns of HO MX and LO MX. The *d*‐spacing calculated from (002) peak is also given; e) TEM f) HRTEM, and g) SAED images of HO MX; h) TEM i) HRTEM, and j) SAED images of LO MX. k) Illustration of the determination of the surface functional group composition. This is based on the O/F ratio from XPS (dashed horizontal line) and comparison of the calculated work functions (triangles, from Ref.[37] and experimental ones (thick black line) for obtaining the O/OH ratio. l) Monte‐Carlo simulation of surface termination distribution.

First, the few‐layered Ti_3_C_2_T_x_ was synthesized from Ti_3_AlC_2_ using a minimally intensive layer delamination (MILD) method.^[^ ^33^ ^]^ After delamination in water, Ti_3_C_2_T_x_ nanosheets with high O‐termination ratio (−O and −OH) and low O‐termination ratio are separated by the gravitational method using centrifugation and denoted as HO MX and LO MX, respectively (**Figure** **1**a, see Supporting Information for details). Ti_3_C_2_T_x_ with more O‐terminations has a higher colloidal stability in water, and thus is more difficult to precipitate under a higher gravitational field and/or a longer centrifuge time.^[^ ^34^ ^]^ Both HO MX and LO MX are stable colloids with a negative zeta potential < −30 mV (Figure S1, Supporting Information). Scanning electron microscopy images show that both MXenes have similar lateral sizes (Figure S2, Supporting Information). The X‐ray photoelectron spectroscopy (XPS) data of the HO MX and LO MX reveals the existence of C, Ti, O, and F, in line with the reported elemental composition from the MILD method (Figure S3, Supporting Information, and see detailed analysis in Tables S1 and S2, Supporting Information).^[^ ^33^, ^35^ ^]^ As shown in Figure 1b, the Ti_3_C_2_ MXene phase is confirmed by the C─Ti bonds in Ti 2p and C 1s, corresponding to the core [TiC_6_] octahedral building blocks.^[^ ^36^ ^]^ The trace amount of TiO_2_ in both samples comes from the partial surface oxidation of MXenes. After removing the contribution from impurities (TiO_2_, organic contaminations, etc.), the Ti atomic percentages and the Ti to surface atom (O and F) ratios were mostly the same in both samples (Figure 1c; Table S3, Supporting Information). However, the O to F atomic ratio in HO MX is 2.12:1 while in LO MX is only 0.82:1.

X‐ray diffraction (XRD) patterns of vacuum‐filtrated films from HO MX and LO MX have identical profiles (Figure 1d), in which high order lamellar peaks can be clearly observed, yielding a *d*‐spacing value of 14.8 Å. Standard deviations of *d*‐spacing calculated from each lamellar peak of HO MX and LO MX are 0.02 and 0.05 Å, respectively (Table S4, Supporting Information), proving thorough delamination of both samples. Transmission electron microscopy (TEM) images show that HO MX and LO MX were single or few‐layered nanosheets (Figure 1e,h). In the high‐resolution TEM (HRTEM), we observe well‐defined crystallinity and hexagonal lattice in both HO MX and LO MX (Figure 1f,i). Selected area electron diffraction (SAED) patterns of both samples (Figure 1g,j) show clear hexagonal diffraction patterns without twinned structure, implying a single layered 2D nanocrystal structure in both HO MX and LO MX. These results prove that Ti_3_C_2_T_x_ with different surface chemistries are yielded.

We further measured their work functions using ultraviolet photoelectron spectroscopy (UPS) (Figure S4, Supporting Information). We indeed observed a higher work function in HO MX (3.82 eV) than in LO MX (2.85 eV), in line with the reported results, where an O‐termination abundant surface leads to a high work function in Ti_3_C_2_T_x_ MXene.^[^ ^37^, ^38^ ^]^ Based on XPS and UPS results (see SI for details), we have identified the surface compositions of MXenes are F_0.55_O_0.12_OH_0.33_ for LO MX and F_0.32_O_0.24_OH_0.44_ for HO MX (Figure 1k). This composition was employed into a Monte‐Carlo simulation using our established cluster expansion model,^[^ ^37^ ^]^ elucidating distributions of O‐(−O and −OH) and F‐terminations. In LO MX with a lower O to F ratio, the regions of O‐terminations were smaller and more discrete (with commonly less than 10 neighboring terminations) than in the HO MXene (with a few tens of neighboring terminations) (Figure 1l; Figure S5, Supporting Information).^[^ ^37^ ^]^

### 2.2 Guided Growth of CoFeLDH on MXene

CoFeLDH‐Ti_3_C_2_T_x_ heterostructures are prepared using an in situ growth method at ambient conditions, as depicted in Supporting Information. The HO MX or LO MX at a fixed weight percentage were used as a template for growing CoFeLDH with Co to Fe atomic feeding ratios of 2 to 1, 3 to 1, 4 to 1, and 6 to 1, resulting in CoFeLDH‐Ti_3_C_2_T_x_ heterostructures. As a control group, CoFeLDH nanosheets were prepared at the same feeding ratios whilst without Ti_3_C_2_T_x_. In total, 12 samples (denotation: Co_n_Fe_1_‐HO MX or LO MX for CoFeLDH‐Ti_3_C_2_T_x_ heterostructures and Co_n_Fe_1_ for CoFeLDH control samples, n = 2, 3, 4, and 6) were synthesized for subsequent investigation. From the inductively coupled plasma optical emission spectroscopy analysis, we found that atomic Co to Fe ratios in the CoFeLDH are consistent with their feeding ratios (Table S5, Supporting Information).

**Figure 2:**
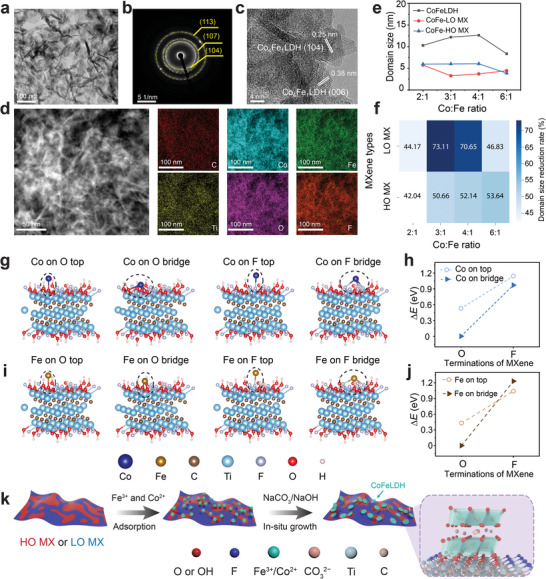
Characterization, and formation mechanism of CoFeLDH‐Ti_3_C_2_T_x_ heterostructures. a) TEM images of Co_4_Fe_1_‐LO MX, and b) corresponding SAED patterns; c) HRTEM image of Co_4_Fe_1_‐LO MX showing nanosheets exposing different crystal planes; d) HAADF‐STEM of Co_4_Fe_1_‐LO MX and associated elemental mapping images of Co_4_Fe_1_‐LO MX nanosheets; e) The domain size of as‐prepared samples calculated using Scherrer equation; f) The domain size reduction rate of CoFeLDH‐Ti_3_C_2_T_x_ compared to CoFeLDH; g) Optimized atomic structures for Co ion adsorbed at different O‐ and F‐terminated sites on MXene (F_0.55_OH_0.38_O_0.19_, average of the HO and LO); h) The binding energy of Co ions at different O‐ and F‐terminated adsorption sites on MXene. i) Optimized atomic structures for Fe ion adsorbed at different O‐ and F‐ terminated sites on MXene. j) The binding energy of Fe ions at different O‐ and F‐terminated adsorption sites on MXene; k) Schematics of the guided growth mechanism of CoFeLDH‐Ti_3_C_2_T_x_ heterostructures.

Next, TEM shows that Co_4_Fe_1_‐LO MX has a “house of cards” structure (**Figure** **2**a–c), consistent with reported LDHs‐MXenes heterostructures obtained through in situ growth or electrostatic assembly.^[^ ^23^, ^25^ ^]^ The SAED pattern shows (104), (107), and (113) diffraction rings of Co_4_Fe_1_ (Figure 2b), indicating a polycrystalline structure. No diffraction ring from the MXene lattice was observed because CoFeLDH is dominated in the heterostructure (ca. 87 wt.%). TEM images (Figure S6, Supporting Information) reveal a consistent 2D nanosheet structure and diffraction rings for Co_4_Fe_1_. Figure 2c shows the lattice fringes with interplanar spacings of 0.25 and 0.38 nm, corresponding to the (104) and (006) crystal planes of Co_4_Fe_1_, respectively. From the high‐angle annular dark‐field scanning transmission electron microscopy (HAADF‐STEM) elemental mapping (Figure 2d) of Co_4_Fe_1_‐LO MX, we confirm the homogenous distribution of C, Co, Fe, Ti, O, and F, indicating heterostructure between the Ti_3_C_2_T_x_ and Co_4_Fe_1_. From TEM images of Co_3_Fe_1_‐HO MX and Co_3_Fe_1_ (Figures S7 and S8, Supporting Information), similar morphologies of CoFeLDH were found, as in Co_4_Fe_1_‐LO MX.

For further phase identification and studying the crystallinity of CoFeLDH‐Ti_3_C_2_T_x_ heterostructures, powder XRD measurements were performed. As shown in Figure S9 (Supporting Information), all the XRD peaks from CoFeLDH and CoFeLDH‐Ti_3_C_2_T_x_ samples can be indexed using CoFeLDH phase (PDF#50‐0235). However, in Co_6_Fe_1_ we observed an impurity Co(OH)_2_ phase, because the positive charge of its cationic layer in the empirical formula [M^2+^_1−x_M^3+^_x_(OH)_2_]^x+^ is not in the stable range of 0.2–0.33.^[^ ^39^ ^]^ The first two diffraction peaks at ca. 11.6° and ca. 23.4° in CoFeLDH can be assigned to the (003) and (006) lamella peaks, respectively. Characteristic lamellar peaks of MXene are absent in heterostructures, consistent with TEM results, indicating that the growth of CoFeLDH on Ti_3_C_2_T_x_ nanosheets inhibits their restacking (Figure 2a–d; Figures S6–S8, Supporting Information). Note that the CoFeLDH lamellar peaks in powder XRD were absent in SAED for both CoFeLDH and CoFeLDH‐Ti_3_C_2_T_x_ samples, indicating that the preferable CoFeLDH nanosheet alignment was parallel to the substrate, as Ti_3_C_2_T_x_.

We then calculated the domain size of LDH phase in CoFeLDH and CoFeLDH‐Ti_3_C_2_T_x_ from the XRD data using the Scherrer equation (Table S6, Supporting Information). Figure 2e shows that the crystalline domain size was the largest in Co_4_Fe_1_ and decreased while lowering the Co to Fe ratio. The mixed phase Co_6_Fe_1_ has the smallest domain size due to Co(OH)_2_ phase formation. Importantly, the presence of Ti_3_C_2_T_x_ has substantially decreased the domain size of CoFeLDH compared to the absence of Ti_3_C_2_T_x_. For Co_3_Fe_1_‐ and Co_4_Fe_1_‐Ti_3_C_2_T_x_ heterostructures, the decrease in domain size was greater in LO MX samples (ca. 70%) than in HO MX samples (ca. 50%) (Figure 2f). For Co_2_Fe_1_‐ and Co_6_Fe_1_‐Ti_3_C_2_T_x_, the domain size decrease was almost identical in LO MX and HO MX samples (≈43% and 50% respectively). These results suggest that the presence of Ti_3_C_2_T_x_ indeed constrains the growth of CoFeLDH, and Co_3_Fe_1_ and Co_4_Fe_1_ nanosheets are sensitive to MXene surface terminations.

For a deeper understanding of how the surface chemistry of Ti_3_C_2_T_x_ affects the growth of CoFeLDH, we used DFT to model Co and Fe ion adsorption on both bridge and top sites of O‐ and F‐terminations, using a Ti_3_C_2_T_x_ surface with composition F_0.55_OH_0.38_O_0.19_ (average of the HO and LO). The calculated binding energies revealed that Co and Fe ions are more energetically favorable binding to O‐terminations than to F‐terminations (Figure 2g–j; Figure S10, Supporting Information). Furthermore, O‐terminations on Ti_3_C_2_T_x_ are more hydrophilic than F,^[^ ^34^ ^]^ making the nucleation and growth of CoFeLDH nanocrystals there easier. Based on both experimental and calculation results, we proposed an O‐termination guided growth mechanism as illustrated in Figure 2k. First, the Co^2+^/Fe^3+^ ions were adsorbed mainly on the O‐terminations on Ti_3_C_2_T_x_ surface. Then, the in situ growth of CoFeLDH was initiated by adding bases such as NaOH and Na_2_CO_3_. Owing to its higher F‐termination ratio, LO MX has a smaller and more discrete O‐rich area compared to HO MX (Figure S10, Supporting Information), leading to smaller crystalline domains in CoFeLDH‐LO MX than CoFeLDH‐HO MX (Figure 2e). Besides, we speculate that the mismatch in lattice parameters between CoFeLDH (hexagonal, *a* = 3.12 Å) and Ti_3_C_2_T_x_ (hexagonal, *a* = 2.99 Å) may also induce lattice distortion, consequently reducing the crystal order and general domain size in the CoFeLDH (Figure S11, Supporting Information).

### 2.3 Electrocatalytic Performance of CoFeLDH‐Ti_3_C_2_T_x_ Toward OER

**Figure 3:**
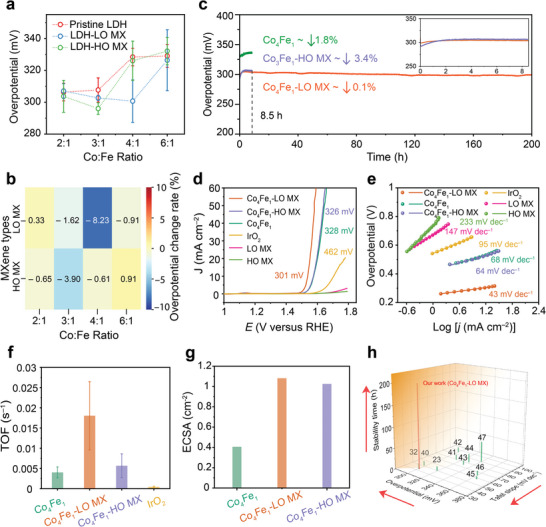
OER activities of the CoFeLDH‐Ti_3_C_2_T_x_ electrocatalyst. a) iR‐corrected average overpotential of different catalysts with three independent measurements at 10 mA cm^−2^ at 5 mV s^−1^ scan rate; b) The change rate of overpotential for CoFeLDH‐Ti_3_C_2_T_x_ heterostructures compared to the pristine CoFeLDH; c) Chronopotentiometry curves of Co_4_Fe_1_, Co_4_Fe_1_‐LO MX, and Co_3_Fe_1_‐HO MX at the current density of 10 mA cm^−2^; d) iR‐corrected OER linear sweep voltammetry curves, with the values indicating overpotentials at 10 mA cm^−2^; e) corresponding Tafel plots of Co_4_Fe_1_‐LO MX, Co_4_Fe_1_‐HO MX, their constituents, and commercial IrO_2_; f) TOF values at the overpotential of 300 mV for Co_4_Fe_1_‐LO MX, Co_4_Fe_1_‐HO MX, and Co_4_Fe_1_ nanosheets, with commercial IrO_2_ as a comparison; g) ECSA at the overpotential of 300 mV for Co_4_Fe_1_‐LO MX, Co_4_Fe_1_‐HO MX, and Co_4_Fe_1_ nanosheets; h) Comparison of overpotential, Tafel slope and stability at 10 mA cm^−2^ of Co_4_Fe_1_‐LO MX (orgin line) with those of previously reported MXene heterostructures OER catalysts (FeNi‐LDH/Ti_3_C_2_‐MXene,^[^ ^32^ ^]^ CoP@3D MXene,^[^ ^40^ ^]^ CoFeLDH/MXene,^[^ ^23^ ^]^ CoFeLDH nanosheet,^[^ ^41^ ^]^ CoFeLDH‐F,^[^ ^42^ ^]^ g‐C_3_N_4_/MXene,^[^ ^43^ ^]^ Co‐LDH@Ti_3_C_2_Tx,^[^ ^44^ ^]^ NiCoS/Ti_3_C_2_T_x_,^[^ ^45^ ^]^ BP QDs/MXene,^[^ ^46^ ^]^ MoSe_2_/MXene.^[^ ^47^ ^]^).

We compared the overpotentials of CoFeLDH at various Co to Fe ratios and their heterostructures with LO and HO MX (**Figure** **3**a; Figures S12 and S13, Supporting Information). The overpotentials of Co_2_Fe_1_ and Co_6_Fe_1_ have minor changes after forming heterostructures with MXene. Among all heterostructures, Co_3_Fe_1_‐HO MX has the lowest overpotential (296 mV) (Figure 3b), although it is not significantly lower than Co_3_Fe_1_ (3.91%). Co_4_Fe_1_‐LO MX achieved the largest overpotential reduction (8.23%) compared to its pristine CoFeLDH. We then studied the stability of samples with low overpotentials, i.e., Co_4_Fe_1_‐LO MX and Co_3_Fe_1_‐HO MX. Surprisingly, Co_4_Fe_1_‐LO MX demonstrates remarkably durable stability with only 0.1% overpotential decay over 200 h chronopotentiometry testing (Figure 3c) and a minor change in the polarization curve (Figure S14, Supporting Information), while the Co_3_Fe_1_‐HO MX and Co_4_Fe_1_ show inferior stability. The overpotential of Co_4_Fe_1_ and Co_3_Fe_1_‐HO MX decayed by 1.8% and 3.4% in 8.5 h, respectively. Thus, we identified Co_4_Fe_1_‐LO MX as the optimal CoFeLDH‐Ti_3_C_2_T_x_ heterostructure, in which a low overpotential and superior stability can be achieved simultaneously.

As the optimized heterostructure, Co_4_Fe_1_‐LO MX exhibits significantly enhanced performance with overpotential of 301 mV at a current density of 10 mA cm^−2^ compared to Co_4_Fe_1_ (328 mV), Co_4_Fe_1_‐HO MX (326 mV) and commercial IrO_2_ (462 mV), while the pristine Ti_3_C_2_T_x_, HO MX and LO MX are not able to operate at 10 mA cm^−2^ (Figure 3d). The highest activity of Co_4_Fe_1_‐LO MX is further corroborated by its OER kinetics (Tafel slope = 43 mV dec^−1^) and intrinsic activity (turnover frequency, TOF values = 0.018 s^−1^ at 300 mV overpotential) (Figure 3e,f). We also observed the smallest charge transfer resistance (*R*_ct_) in Co_4_Fe_1_‐LO MX from the electrochemical impedance spectroscopy results among all samples (Figure S15, Supporting Information), aligning with its minimal Tafel slope. The electrochemically active surface area (ECSA) of the catalysts was determined based on the double‐layer capacitance (*C*_dl_). As illustrated in Figure 3g and Figure S16 (Supporting Information), the Co_4_Fe_1_‐LO MX catalyst exhibits the highest *C*_dl_ value of 0.618 mF cm^−2^, significantly exceeding that of Co_4_Fe_1_ (0.232 mF cm^−2^). Consequently, the Co_4_Fe_1_‐LO MX catalyst possesses the largest ECSA of 1.08 cm^2^, surpassing that of Co_4_Fe_1_ (0.41 cm^2^), implying more active sites present in Co_4_Fe_1_‐LO MX. This observation is another strong evidence, validating our guided growth assumption. Co_4_Fe_1_‐LO MX has small crystalline domains with a high number of active sites when growing via MXene surface with more discrete O‐termination regions. To validate the potential for large‐scale production, we also scaled up the synthesis by a factor of ten. Only a 3 mV increase in overpotential was observed in the large‐scale sample compared to the small‐scaled sample (Figure S17, Supporting Information), indicating a good scalability. When considering the overpotential at 10 mA cm^−2^, the Co_4_Fe_1_‐LO MX catalyst demonstrates remarkably competitive performance metrics compared to existing catalysts of its kind, where the highest activity and stability were achieved simultaneously (Figure 3h; Table S7, Supporting Information).

### 2.4 Mechanism of Electrocatalysis in CoFeLDH‐Ti_3_C_2_T_x_

From XRD results and the electrocatalytic performance, the reduced domain size in CoFeLDH‐Ti_3_C_2_T_x_ heterostructures translates to more active sites than pure LDHs.^[^ ^48^ ^]^ However, the superior intrinsic activity of these catalysts, evidenced by their lower overpotential and higher TOF values, cannot be solely attributed to the augmented number of active sites. Underlying mechanisms contributing to their enhanced performance need further investigation.

**Figure 4:**
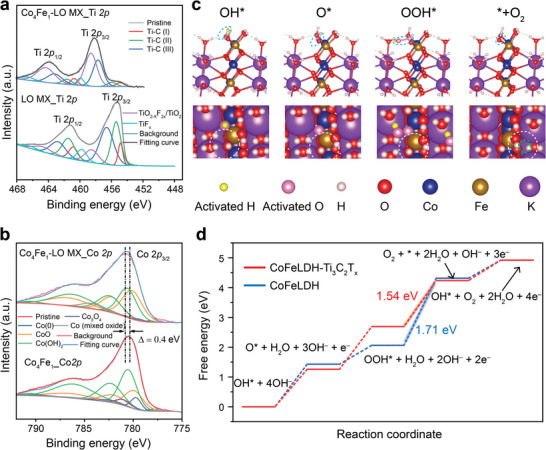
Analysis of the catalytic mechanism for OER. a) Comparison of Ti 2p spectra of LO MX and Co_4_Fe_1_‐LO MX; b) Comparison of Co 2p spectra for Co_4_Fe_1_ and Co_4_Fe_1_‐LO MX; c) Side and top view atomic structures during OER steps in the case of CoFeLDH‐Ti_3_C_2_T_x_ with an electron added to the system. Blue/white circles denote adsorbates on the side/top views. OER intermediates are color‐coded for clarity, with yellow representing hydrogen and rose indicating oxygen (in place of white and red, respectively). A dashed green circle signifies the formation of a surface O vacancy. Reaction centers are highlighted by large white circles; d) Reaction free‐energy diagrams for OER on the pristine CoFeLDH and CoFeLDH‐Ti_3_C_2_T_x_ with rate‐limiting steps highlighted.

XPS analysis of Ti, Co, Fe, C, O, and F was conducted on selected CoFeLDH and CoFeLDH‐Ti_3_C_2_T_x_ heterostructures to unveil their composition and electron transfer mechanisms (Figures S18 and S19, Supporting Information). Compared to pristine HO/LO MX, we observed an increased fraction in the TiO_x_ species and a decreased fraction in the C─Ti bond in both Ti 2p spectra of Co_4_Fe_1_‐HO/LO MX and Co_3_Fe_1_‐HO/LO MX, (**Figure** **4**a; Figures S20 and S21, Supporting Information), implying the oxidation of Ti in the heterostructures. The O 1s spectra, dominated by CoFeLDH surface OH groups, remained similar in Co_3_Fe_1_, Co_4_Fe_1_, and their Ti_3_C_2_T_x_ heterostructures (Figures S22 and S23, Supporting Information). By deconvoluting the Co 2p_3/2_ peak using the method reported by Biesinger et al.,^[^ ^49^ ^]^ CoO and Co(OH)_2_ are the major forms of Co (Figure 4b; Figures S24 and S25 and Table S8, Supporting Information), consistent with previous reports.^[^ ^49^ ^]^ The high‐resolution spectra of Co_4_Fe_1_‐HO/LO MX reveal a slight positive shift (≈0.4 eV) in the Co 2p peak toward higher binding energy (Figure 4b; Figure S24, Supporting Information), indicating the reduction of Co. No such shift was found in Co_3_Fe_1_‐HO/LO MX (Figure S25, Supporting Information). On the other hand, the Fe 2p peaks of all CoFeLDH‐Ti_3_C_2_T_x_ heterostructures exhibit a negative shift (≈0.3–0.4 eV) compared to their CoFeLDH counterparts, implying the reduction of Fe (Figure S26, Supporting Information). Our findings suggest that electron transfer pattern changes in Co_4_Fe_1_‐HO/LO MX and Co_3_Fe_1_‐HO/LO MX heterostructures owing to their structural difference.^[^ ^23^, ^49^, ^50^ ^]^ The transfer is from Ti to Co and Fe in the former one, while primarily occurs from Ti to Fe rather than Co in the latter one.

For a better understanding of the stable catalyst structure and active sites, Co_4_Fe_1_‐LO MX after extended catalytic operation was also studied. The O 1s spectrum remained nearly unchanged (Figure S27, Supporting Information), suggesting minimal alteration of surface OH groups on the CoFeLDH. Conversely, Co 2p spectra revealed a dramatic increase in Co_3_O_4_ concentration (from 2.62% to 68.03%), coupled with a significant decrease in CoO and Co(OH)_2_ (Figure S28 and Table S8, Supporting Information). This shift implies the oxidation of Co^2+^ to Co^3+^ during OER under applied potential, a characteristic transformation associated with the γ‐phase CoFeLDH formation, as confirmed by the XRD pattern (Figure S29, Supporting Information).^[^ ^16^ ^]^ Contrary to reports assuming that Ti_3_C_2_T_x_ is stable under electrocatalysis,^[^ ^23^ ^]^ Ti_3_C_2_T_x_ in Co_4_Fe_1_‐LO MX inevitably undergoes oxidation and degradation during alkaline OER, consistent with findings by Kuznetsov et al.^[^ ^51^ ^]^ As evidenced by our XPS data, fluorine and oxygen‐doped graphite carbon (C_F/O_), and hydrated TiO_2_,^[^ ^52^ ^]^ are formed from Ti_3_C_2_T_x_ (Figures S28 and S30, Supporting Information). Therefore, the stable reconstructed catalyst is γCoFeLDH‐TiO_2_‐C_F/O_ and the γCoFeLDH is the actual catalytic active phase. The restructured TiO_2_ and C_F/O_ matrices potentially contribute to the increased stability.^[^ ^13^ ^]^ Additionally, the smaller domain size along the (003) direction indicates a decreasing number of layers in LDH, which significantly enhances the stability of the catalyst for electrocatalytic OER.^[^ ^53^ ^]^

Based on our experimental results of the electron transfer pattern and active sites, we performed a DFT simulation to elucidate the catalytic mechanism at the atomic level as previously reported.^[^ ^15^, ^16^ ^]^ The stable γCoFeLDH phase was chosen, and the OER pathways were investigated with or without additional electrons. Specifically, the OER performance is assessed utilizing the (0 1 $\bar{10}$) surface of γCoFeLDH with K and H_2_O intercalated between layers and the CoFeLDH edge terminated by OH, as shown in Figure 4c and Figure S31 (Supporting Information). The surrounding water is considered via the continuum solvation model. According to the Mars van Krevelen mechanism under OER conditions, OER on these surfaces is initiated with the deprotonation of surface OH (OH^*^ to O^*^).^[^ ^15^, ^16^, ^54^ ^]^ We then calculated the free energy step diagram (4OH^−^ + OH^*^ → 3OH^−^+ O^*^ + H_2_O + e^−^ → 2OH^−^ + OOH^*^ + H_2_O + 2e^−^ → O_2_ + 2H_2_O + 3e^−^ + * + OH^−^ → OH^*^ + O_2_ + 2H_2_O + 4e^−^) for pristine CoFeLDH and for CoFeLDH‐MX containing additional electrons (Tables S9–S12, Supporting Information). Figure 4d presents diagrams for both cases, alongside their corresponding atomic structures in Figure 4c and Figure S31 (Supporting Information). Pristine CoFeLDH results closely match previous reports.^[^ ^16^ ^]^ Importantly, the rate‐determining step in CoFeLDH is the conversion of OOH^*^ to O_2_. Donating electrons to γCoFeLDH elevates the energy of OOH^*^, while leaving others unchanged. This reduces the free energy of the rate‐determining step from 1.71 to 1.54 eV, thus explaining the reduction in overpotential. We also employed an alternative modeling approach by substituting potassium with calcium to increase the electron density in the system, which also shows a decreased overpotential (Figure S32 and Tables S13 and S14, Supporting Information), consistent with our experimental findings, albeit with a smaller change. Comparison of the Bader charges for systems with and without additional electron (Tables S15–S17, Supporting Information) confirms that the electron density has increased in CoFeLDH‐Ti_3_C_2_T_x_ heterostructures.

## 3 Conclusion

In summary, we presented a guided growth strategy and mechanism investigation of CoFeLDH on Ti_3_C_2_T_x_ surface, leading to heterostructures with superior OER activity and stability. Assisted by Monte‐Carlo simulation and DFT calculations, we identified that the O‐terminations on Ti_3_C_2_T_x_ enable guided growth of CoFeLDH. The Ti_3_C_2_T_x_ surface with small and discrete O‐terminated regions leads to small CoFeLDH nanocrystals, with the most dramatic domain size decrease seen in the cases of Co_3_Fe_1_ and Co_4_Fe_1_ on Ti_3_C_2_T_x_. Interestingly, Co to Fe atomic ratios in CoFeLDH do not only affect the domain size in heterostructures but also change the electron transfer pattern between two components. For instance, Ti in Ti_3_C_2_T_x_ transfers electrons to both Co and Fe in Co_4_Fe_1_‐Ti_3_C_2_T_x_ heterostructures, but only to Fe in Co_3_Fe_1_‐Ti_3_C_2_T_x_ heterostructures. As a result, LO MX remarkably improved the intrinsic activity of Co_4_Fe_1_ with a lower overpotential of 301 mV at 10 mA cm^−2^, a low Tafel slope of 43 mV dec^−1^, a high TOF value of 0.018 s^−1^, and robust stability at 10 mA cm^−2^ with only 0.1% decay for 200 h. The high performance is maintained in the ×10 scaling up products. Overall, the Co_4_Fe_1_‐LO MX exhibits superior OER performance among the reported LDH‐MXene heterostructures. Our experimental results indicate that the exceptional stability of CoFeLDH‐Ti_3_C_2_T_x_ heterostructures resulted from the formation of stable γCoFeLDH on the TiO_2_ and C_F/O_ matrices reconstructed from Ti_3_C_2_T_x_, as well as the decreased thickness of CoFeLDH due to the guided growth mechanism of MXene terminations. DFT calculations reveal that Ti₃C₂T_x_ induces charge redistribution in CoFeLDH, which reduces the rate‐limiting energy barrier for OER and enhances the catalytic activity.

Compared to the previous works that mostly focused on simple chemical compositions and interfacial coupling, our work has made substantial progress in understanding the rich surface chemistry of MXenes, various compositions of LDH, and charge‐transfer/reconstruction at the LDH‐MXene interface with both experiments and calculations. We have listed the detailed comparison in Table S22 (Supporting Information). However, to further maximize the potential of LDH‐MXene materials for electrocatalysis and other applications, more effort should be put into fundamental studies, such as developing new structures from novel synthetic routes, investigating the chemical composition freedom from both LDHs and MXenes, and characterization with in situ/operando techniques, etc.

## Conflict of Interest

The authors declare no conflict of interest.

## Supporting information



Supporting Information

## Data Availability

The data that support the findings of this study are available from the corresponding author upon reasonable request.
