# Supplementary material for: Guided Heterostructure Growth of CoFe LDH on Ti3C2Tx MXene for Durably High Oxygen Evolution Activity
Source: Small. 2024 Sep 10;21(3):2404927. doi: 10.1002/smll.202404927 (PMC11753486; doi:10.1002/smll.202404927)
Supplement: Supplementary file 1 — Supporting Information [file SMLL-21-2404927-s001.pdf]

NANO · MICRO
small

Supporting Information

for *Small*, DOI 10.1002/smll.202404927

Guided Heterostructure Growth of CoFe LDH on $Ti_3C_2T_x$ MXene for Durably High Oxygen Evolution Activity

Jiali Sheng, Jiahui Kang, Pan Jiang, Kristoffer Meinander, Xiaodan Hong, Hua Jiang, Nonappa, Olli Ikkala, Hannu-Pekka Komsa, Bo Peng* and Zhong-Peng Lv**

Supporting information

Guided heterostructure growth of CoFe LDH on $Ti_3C_2T_x$ MXene for durably high oxygen evolution activity

Jiali Sheng[a], Jiahui Kang[a], Pan Jiang[a,b], Kristoffer Meinander[c], Xiaodan Hong[a], Hua Jiang[a], Nonappa[d], Olli Ikkala[a], Hannu-Pekka Komsa[e,*], Bo Peng[a,f,*], Zhong-Peng Lv[a,*]

a. Department of Applied Physics, Aalto University, Aalto FIN-00076, Finland

E-mail: zhongpeng.lyu@aalto.fi

b. Research Institute of Wood Industry, Chinese Academy of Forestry, Xiangshan Road, Beijing 100091, China

National Engineering Research Center for Low-carbon and Efficient Utilization of Forest Biomass, Xiangshan Road, Beijing 100091, China

c. Department of Bioproducts and Biosystems, Aalto University, Aalto FIN-00076, Finland

d. Faculty of Engineering and Natural Sciences, Tampere University, Tampere, FI-33101 Finland

e. Microelectronics Research Unit, Faculty of Information Technology and Electrical Engineering, University of Oulu, FIN-90014 Oulu, Finland

E-mail: Hannu-Pekka.Komsa@oulu.fi

f. Department of Materials Science, Advanced Coating Research Center of Ministry of Education of China, Fudan University, Shanghai 200433, China

E-mail: peng_bo@fudan.edu.cn

Contents

Supporting Methods 4

Preparation of $Ti_3C_2T_x$ and CoFeLDH-$Ti_3C_2T_x$ 4

Structural characterization of $Ti_3C_2T_x$ and CoFeLDH-$Ti_3C_2T_x$ nanosheets 4

Electrochemical measurements 5

Monte-Carlo simulations 6

Density-functional theory calculations 7

Supporting Figures 9

Fig. S1 Zeta potentials of HO MX and LO MX. 9

Fig. S2 SEM images of two different MXenes. 9

Fig. S3 XPS survey spectra of HO MX and LO MX. 10

Fig. S4 Ultraviolet photoelectron spectroscopy of HO MX and LO MX. 10

Fig. S5 Distribution area of −O/−OH of HO/LO MX obtained from Montecarlo simulation. 11

Fig. S6 Structural characterization of Co_4Fe_1 11

Fig. S7 Structural characterization of Co_3Fe_1-HO MX. 12

Fig. S8 Structural characterization of Co_3Fe_1 13

Fig. S9 XRD patterns of samples. 13

Fig. S10 DFT calculations for binding energy of Co and Fe absorb on MXene surface under vacuum. 14

Fig. S11 Proposed mechanism of domain size decrease of CoFeLDH caused by lattice mismatch. 14

Fig. S12 iR-corrected average overpotential of as-prepared catalysts at 10 mA cm^{-2} at 5 mV/s scan rate. 15

Fig. S13 OER performance for Co_3Fe_1 and related samples. 15

Fig. S14 LSV curves of Co_4Fe_1-LO MX before and after 200 h chronopotentiometry test. 16

Fig. S15 EIS spectra of various catalysts toward OER. 16

Fig. S16 CV curves and Cdl of Samples. 17

Fig. S17 Scaled-up synthesis of Co_4Fe_1-LO MX by 10 times. 17

Fig. S18 XPS survey spectra of Co_4Fe_1, Co_4Fe_1-LO MX, and Co_4Fe_1-HO MX. 18

Fig. S19 XPS survey spectra of Co_3Fe_1, Co_3Fe_1-LO MX, and Co_3Fe_1-HO MX. 18

Fig. S20 Comparision of Ti 2p and F 1s spectra in Co_4Fe_1-MX samples. 19

Fig. S21 Comparision of Ti 2p and F 1s spectra in Co_3Fe_1-MX samples. 19

Fig. S22 Comparision of C 1s and O 1s spectra in Co_4Fe_1-MX samples. 20

Fig. S23 Comparision of C 1s and O 1s spectra in Co_3Fe_1-MX samples. 20

Fig. S24 Comparison of Co 2p spectra in pristine Co_4Fe_1 and Co_4Fe_1-HO MX. 21

Fig. S25 Comparison of Co 2p spectra in Co_3Fe_1-MX samples. 21

Fig. S26 Comparison of Fe 2p spectra of samples. 22

Fig. S27 XPS spectrum of Co_4Fe_1-LO MX for O 1s after stability test. 23
Fig. S28 Post-catalytic XPS characterization of Co_4Fe_1-LO MX. 23
Fig. S29 Post-catalytic XRD characterization of Co_4Fe_1-LO MX. 23
Fig. S30 Post-Catalytic XPS Characterization of Co_4Fe_1-LO MX with C 1s Spectra Analysis... 24
Fig. S31 Free energy profiles of pristine γ-CoFeLDH (model 1) for OER. 25
Fig. S32 Free energy profiles of CoFeLDH-$Ti_3C_2T_x$ (model 2) for OER. 26
Supporting Tables 27
Table S1 XPS analysis of LO MX. 27
Table S2 XPS analysis of HO MX. 28
Table S3 Atomic concentrations (atomic-%) and the O to F ratios in HO MX and LO MX. 29
Table S4 Calculated *d*-spacing from each lamellar peak in HO MX and LO MX. 29
Table S5 The atomic ratio of Co/Fe for as-prepared samples rely on the ICP-OES analysis. 30
Table S6 Domain size calculated from XRD results. 31
Table S7 Overpotential, Tafel slope and stability at 10 mA cm^{-2} of Co_4Fe_1LDH-LO MX and previously reported MXene heterostructures. 32
Table S8 Relative concentrations of the different components of cobalt. 33
Table S9 DFT calculated free energy contributions for all configurations of pristine CoFeLDH. 33
Table S10 Reaction Gibbs free energy of pristine CoFeLDH. 33
Table S11 DFT calculated free energy contributions for all configurations of CoFeLDH-$Ti_3C_2T_x$ model 1 with one added electron. 34
Table S12 Reaction Gibbs free energy of model 1. 34
Table S13 DFT calculated free energy contributions for all configurations of CoFeLDH-$Ti_3C_2T_x$ model 2 with one K replaced by Ca. 34
Table S14 Reaction Gibbs free energy of model 2 35
Table S15 Bader charge of active atoms for pristine CoFeLDH. 35
Table S16 Bader charge of active atoms for CoFeLDH-$Ti_3C_2T_x$ model 1. 35
Table S17 Bader charge of active atoms for CoFeLDH-$Ti_3C_2T_x$ model 2. 35
Table S18 DFT calculated magnetic moments of active atoms for pristine CoFeLDH. 36
Table S19 DFT calculated magnetic moments of active atoms for CoFeLDH-$Ti_3C_2T_x$ model 1. 36
Table S20 DFT calculated magnetic moments of active atoms for CoFeLDH-$Ti_3C_2T_x$ model 2. 36
Table S21 Numerical values for free energy contributions to H_2 and H_2O molecules. 37
Table S22 The detailed comparison table of the highlights (excluding catalytic performance and mechanism) of this work with other similar works. 38
Reference 39

Supporting Methods

Preparation of $Ti_3C_2T_x$ and CoFeLDH-$Ti_3C_2T_x$

Preparation of few layered $Ti_3C_2T_x$. LiF (2 g, powder 300 mesh, Sigma Aldrich) was mixed with 5 mL of deionized (DI) water and 30 mL of 12M HCl (37%, Merk) in a 100 mL PP bottle under magnetic stirring at 35 °C for 5 min until a clear solution was formed. Then Ti_3AlC_2 MAX (2 g, powder 325 mesh) dispersed in 5 mL of DI water (Millipore system, 18.2 MW cm) was added dropwise to the above solution in a course of 10 min. After 24 h, the sediment was separated by centrifuge at 3500 rpm for 5 min in a 50 mL tube. Using DI water to wash the sediment for a few times until a slurry was formed when pH close to 6. After adding 35 mL DI water to the slurry and vortex for 1 h, the supernatant contains O-rich few layered $Ti_3C_2T_x$ was collected by 60 min centrifuge at 3500 rpm, labeled as HO MX. Add 35 mL DI water to the residual slurry and vortex for another hour, collect the supernatant contains less O-terminated few layered $Ti_3C_2T_x$ by 30 min centrifuge at 3500 rpm, labeled as LO MX. The two dispersions were store it at 5°C under N_2. The concentration was obtained using gravimetric method by weighing certain volume of fully dried dispersion.

Preparation of CoFeLDH-$Ti_3C_2T_x$ and CoFeLDH nanosheets. In a typical synthesis, $Co(NO_3)_2{\cdot}6H_2O$ (145.5 mg, 98%, Sigma Aldrich) and $FeCl_3{\cdot}6H_2O$ (33.8 mg, 99%, Sigma Aldrich) were dissolved in DI water (degassed, 5 mL) to form a homogeneous solution (solution A, Co : Fe = 4:1). Meanwhile, solution B contains Na_2CO_3 (39.7 mg, 99.8%, Honeywell) and NaOH (105 mg, 99.8%, Fisher Scientific) dissolved in 5 mL degassed DI water was prepared. At RT, solutions A and B were simultaneously added dropwise into 10 mL degassed DI water containing 4.75 mg LO MX. After stirring at 400 rpm for 24 h, the sediment was collected by centrifugation, and then washed 4 times with 30 mL of DI water. Finally, adding 6 mL H_2O to redisperse the product, labeled as Co_4Fe_1-LO MX, then store at 5°C under N_2. Other samples were prepared by changing the Co to Fe ratio from 2:1 to 6:1, or different MXenes, where the mass ratio of MXene to the total mass of Co and Fe is 1:7.7. CoFeLDH nanosheets with different Co to Fe ratios were prepared by the same method except no MXene was used.

Structural characterization of $Ti_3C_2T_x$ and CoFeLDH-$Ti_3C_2T_x$ nanosheets

The morphologies of as-prepared samples were characterized by transmission electron microscopy (TEM, JEOL JEM-2200FS). High resolution TEM (HRTEM), selected area

electron diffraction (SAED) patterns, high-angle annular dark-field scanning transmission electron microscopy (HAADF-STEM) and scanning transmission electron microscopy-energy dispersive X-ray spectroscopy (STEM-EDX) element mappings were recorded on JEM-2200FS systems at an accelerating voltage of 200 kV.

The contents of Co and Fe were obtained by inductively coupled plasma optical emission spectroscopy (ICP-OES) measurement performed on an Agilent 730 series ICP optical emission spectrometer.

Powder XRD patterns were collected on a Rigaku SmartLab X-Ray diffraction with Cu Kα radiation (λ = 0.15406 nm) at 35 kV 20 mA at a scanning rate of 3° min^{-1} in a 2θ angle range from 30° to 70° to study crystal structure. The average domain size can be estimated with the Scherrer equation:[1]

$$D_{hkl} = \frac{K\lambda}{B_{hkl}\cos\theta} \quad (1)$$

Where D_{hkl} is the crystallite size in the direction perpendicular to the lattice planes, hkl are the Miller indices of the planes being analyzed, and K is a numerical factor frequently referred to as the crystallite-shape factor (we use 0.90 here). λ is the wavelength of the X-rays, B_{hkl} is the full width at half-maximum (FWHM) of the X-ray diffraction peak in radians and θ is the Bragg angle.

X-ray photoelectron spectroscopy (XPS) spectra were recorded on a Kratos Axis Ultra system to investigate the chemical state of related samples. The XPS spectra were charge-corrected relative to the position of C-C bonding of carbon at 284.8 eV. CasaXPS software was used for all the XPS analyses.

Electrochemical measurements

Electrochemical measurements were carried out on a standard three-electrode system (Metrohm Autolab PGSTAT302N potentiostat) at room temperature in 1.0 M KOH with continuously N_2 purification. A Hg/HgO electrode (1.0 M KOH), a graphite rod, and a glass carbon (GC) electrode were used as the reference electrode, the counter electrode, and the working electrode, respectively. A certain amount of the catalyst ink was dropped on the polished GC electrode with a loading mass of 0.2 mg cm^{-2} and then dried at room temperature. Subsequently, 3 μL of Nafion solution (vol(isopropanol) : vol(5 wt% Nafion) : vol(H_2O) = 85:5:10) was dropped on the above electrode and dried at room temperature. For stability test

and post-catalytic characterization of XPS, the catalyst inks were drop-dried at room temperature on carbon paper (1 × 0.2 cm^2) with a loading mass of 0.2 mg cm^{-2}.

The linear sweep voltammetry (LSV) curves were collected at 5 mV s^{-1}. Electrochemical impedance spectroscopy (EIS) measurements were conducted in the range of 10 k Hz to 0.1 Hz with a 5 mV amplitude at 1.33 V vs. RHE. Durability tests were conducted through chrono-potentiometric methods (10 mA cm^{-2}). All potentials were converted to voltage with reference to the reversible hydrogen electrode (RHE): $E_{RHE} = E_{Hg/HgO} + 0.098 + 0.059 \times pH$. The overpotential (η) was calculated according to the following equation: η = E vs. RHE −1.23 V. All polarization curves were iR-corrected, where R is uncompensated electrolyte resistance (R) measured by electrochemical impedance spectroscopy. The electrochemical double-layer capacitance (C_{dl}) was measured by Cyclic voltammograms (CVs) between 0.94 and 1.04 V vs. RHE at the scan rates from 20 mV s^{-1} to 40 mV s^{-1} without any Faradaic processes involved. The ECSA of a catalyst sample is calculated from the double-layer capacitance according to the equation 2:

$$ECSA = \frac{C_{dl}}{C_s} \quad (2),$$

where Cs is the specific capacitance value (40 μF cm^{-2}) for flat standard with 1 cm^2 of atomically smooth planar surface.[2]

The turnover frequency (TOF) values were calculated based on the assumption that all metal atoms are active sites (lower TOF limits were calculated) according to the following equation 4:[3,4]

$$TOF = \frac{J \cdot A}{4F \cdot n} \quad (3)$$

Here, *J* represents the current density normalized by geometric area of electrode at the 300 mV of overpotential, A stands for geometric area of electrode, the number 4 means 4 electron transfer per mole of O_2, F is Faraday's constant (96485.3 C mol^{-1}), n is the moles of metal atoms on the electrode, which was obtained from ICP-OES results.

Monte-Carlo simulations

The cluster expansion model for describing the interactions between the functional groups and the Monte Carlo simulation details are taken from Ref.[5] In particular, the calculations were carried out using ATAT software,[6–8] 40 × 40 supercell, and the simulation temperature gradually reduced from 5300 K to 300 K.

The surface composition used in the simulations was obtained by first taking the O/F composition from XPS, where the O can be either −O or −OH group, and then finding OH concentration with the calculated work function matching with the experimental one from UPS experiments. This is illustrated in **Fig. S9**. The final compositions used in the calculations are $F_{0.32}O_{0.24}OH_{0.44}$ for HO MX and $F_{0.55}O_{0.12}OH_{0.33}$ for LO MX.

Density-functional theory calculations

Our computational approach largely follows that described by Dionigi *et al.* in Refs.[9,10] We adopt the proposed structural models for the the γ-CoFe bulk (in Co_3Fe_1 stoichiometry) and surfaces, and the same computational parameters. In detail, we use the optPBE functional,[11] 500 eV plane-wave cutoff, Hubbard U-corrections of 3.50 eV and 2.56 eV for Co and Fe, respectively, and 1 × 4 × 3 k-point mesh (3 k-points perpendicular to the layers and 4 k-points along the lateral periodic direction). All calculations are carried out using projector-augmented waves framework as implemented in VASP.[12–14]

The reaction steps are obtained from Gibbs free energy differences between the products and reactants, e.g. for the first H desorption step (OH* → O*):

$$\delta G(\mathrm{OH^*\rightarrow O^*}) = G(\mathrm{O^*}) - [G(\mathrm{OH^*}) - \mu_\mathrm{H}]$$

$$\delta G(\mathrm{OH^*\rightarrow OOH^*}) = G(\mathrm{OOH^*}) - [G(\mathrm{OH^*}) + \mu_\mathrm{O}]$$

$$\delta G(\mathrm{OH^*\rightarrow V+O_2}) = G(\mathrm{V}) - [G(\mathrm{OH^*}) - \mu_\mathrm{OH}] + 4.92\ \mathrm{eV}$$

$$\delta G(\mathrm{OH^*\rightarrow OH^*+O_2}) = G(\mathrm{OH^*}) - [G(\mathrm{OH^*})] + 4.92\ \mathrm{eV}$$

Standard electrode potential for formation of O_2 from water is 1.23 eV with respect to standard hydrogen electrode and involves four electrons, thus yielding the energy 4 × 1.23 eV = 4.92 eV.

The Gibbs free energy includes the vibrational contributions from the active molecule (the 0-3 atoms adsorbed on the bridge site) and the solvation correction:

$$G = E_{DFT} + E_{ZPE} + \delta H - TS + E_{sol}$$

The solvation energy correction is estimated using implicit solvation model as implemented in the VASPsol.[15] This is the main difference between our calculations and those of Dionigi *et al.* [10] On one hand, the solvation shell is more accurately described using explicit water molecules. On the other hand, implicit solvation avoids sensitivity to the exact placement of

the water molecules and thus well suited for studying small energy changes due to electron donation in this work. The numerical values of total energies and free energy corrections for the three models are listed in **Tables S9**, **S11**, and **S13**.

The chemical potentials of H and O in aqueous solution are calculated as follows.

$$\mu_{\mathrm{H}} = \frac{E(\mathrm{H_2})}{2} + E_{\mathrm{ZPE}} + \delta H - TS + [-kT\ln(10)\,pH + eU]$$

$$\mu_{\mathrm{H_2O}} = E(\mathrm{H_2O}) + E_{\mathrm{ZPE}} + \delta H - TS$$

We note that the free-energy diagrams are given with respect to the reversible hydrogen electrode (as in Refs.[9,10]), which cancels out the pH dependence in μ_{H} and consequently in μ_{OH}.

The chemical potentials for OH and O are then obtained from equilibrium with H_2O as

$$\mu_{\mathrm{OH}} = \mu_{\mathrm{H_2O}} - \mu_{\mathrm{H}}$$

$$\mu_{\mathrm{O}} = \mu_{\mathrm{H_2O}} - 2\mu_{\mathrm{H}}$$

We are not using the O_2 reference owing to the difficulties DFT has in describing the O_2 molecule. The E and E_{ZPE} are calculated using the same computational parameters as for the LDH calculations. For δH and S we adopt experimental values listed in the NIST-JANAF thermochemical tables,[16] which already account for the solvation effects. All values are listed in **Table S21**.

The magnetic moments of relevant atoms were also extracted from the calculations, with the values for the three models listed in **Table S18−S20**.

The calculation details for the Co and Fe ion adsorption on the MXene surface are chosen to be largely consistent with the above. We use optPBE functional, 500 eV plane-wave cutoff, and the same Hubbard U corrections for Co and Fe. The MXene model was created using the same cluster expansion as used in the Monte-Carlo simulations, but choosing the average surface composition between HO- and LO-MXenes, $F_{0.55}O_{0.38}OH_{0.19}$, and a 4x4 supercell size. Model of this size contains sufficient variety of local adsorption sites, both F-rich and O-rich environments, yet still allowing us to keep the sane Fermi-level for more reliable comparison among the adsorption sites. Brillouin zone was sampled using 4x4 k-point mesh. Finally, we accounted for the solvation via a continuum model as above, but did not evaluate the vibrational free energy corrections.

Supporting Figures

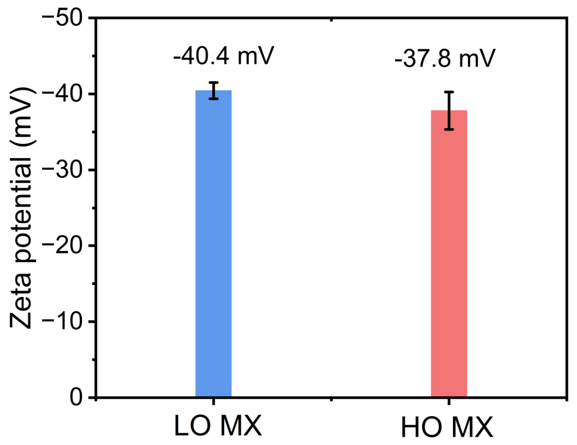

Fig. S1 Zeta potentials of HO MX and LO MX. Error bars represent the standard deviation obtained from 3 independent measurements.

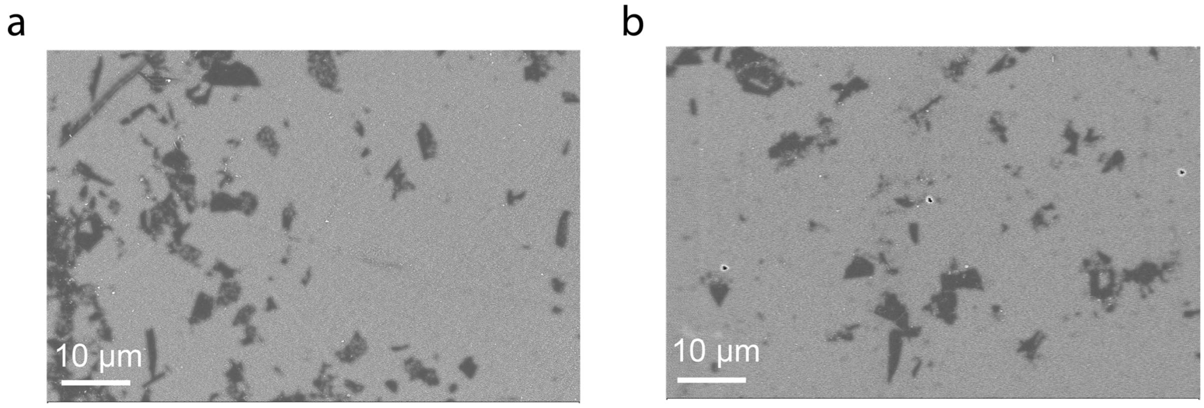

Fig. S2 SEM images of two different MXenes. (**a**) LO MX and (**b**) HO MX on the AAO substrate.

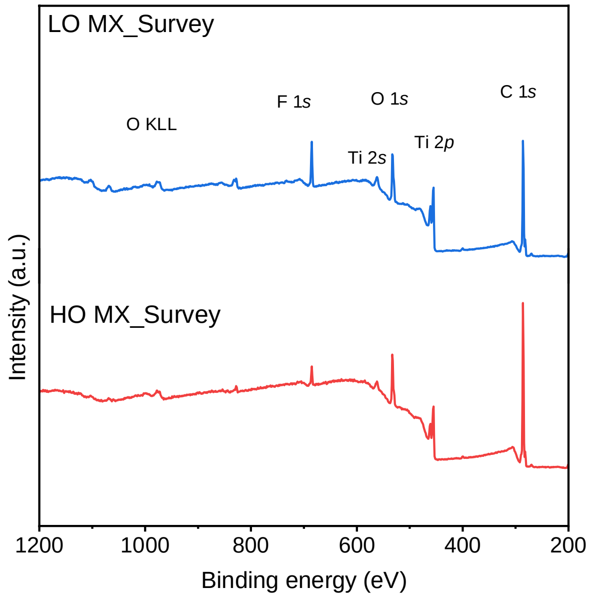

Fig. S3 XPS survey spectra of HO MX and LO MX.

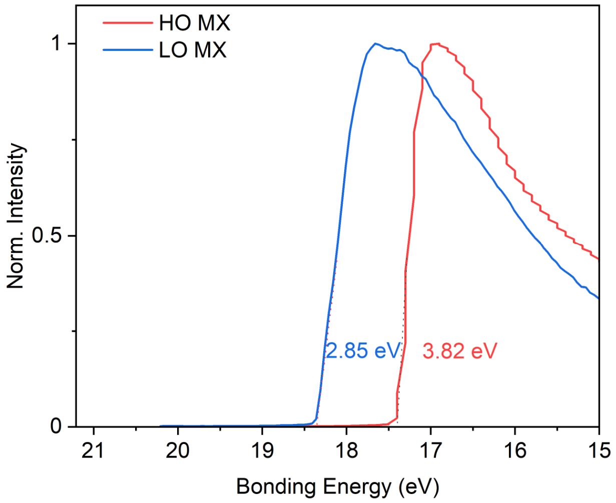

Fig. S4 Ultraviolet photoelectron spectroscopy of HO MX and LO MX.

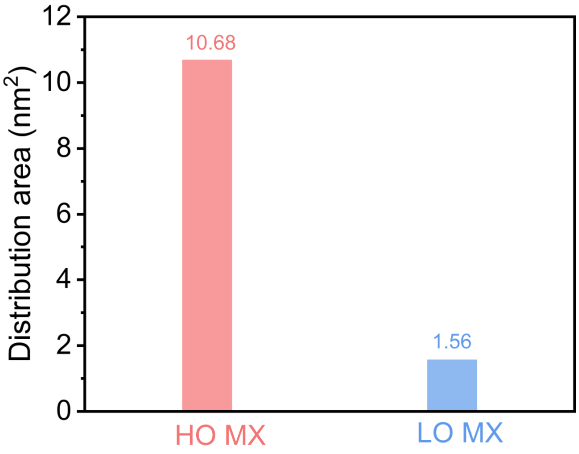

Fig. S5 Distribution area of −O/−OH of HO/LO MX obtained from Montecarlo simulation. The total area selected is 544.39 nm^2.

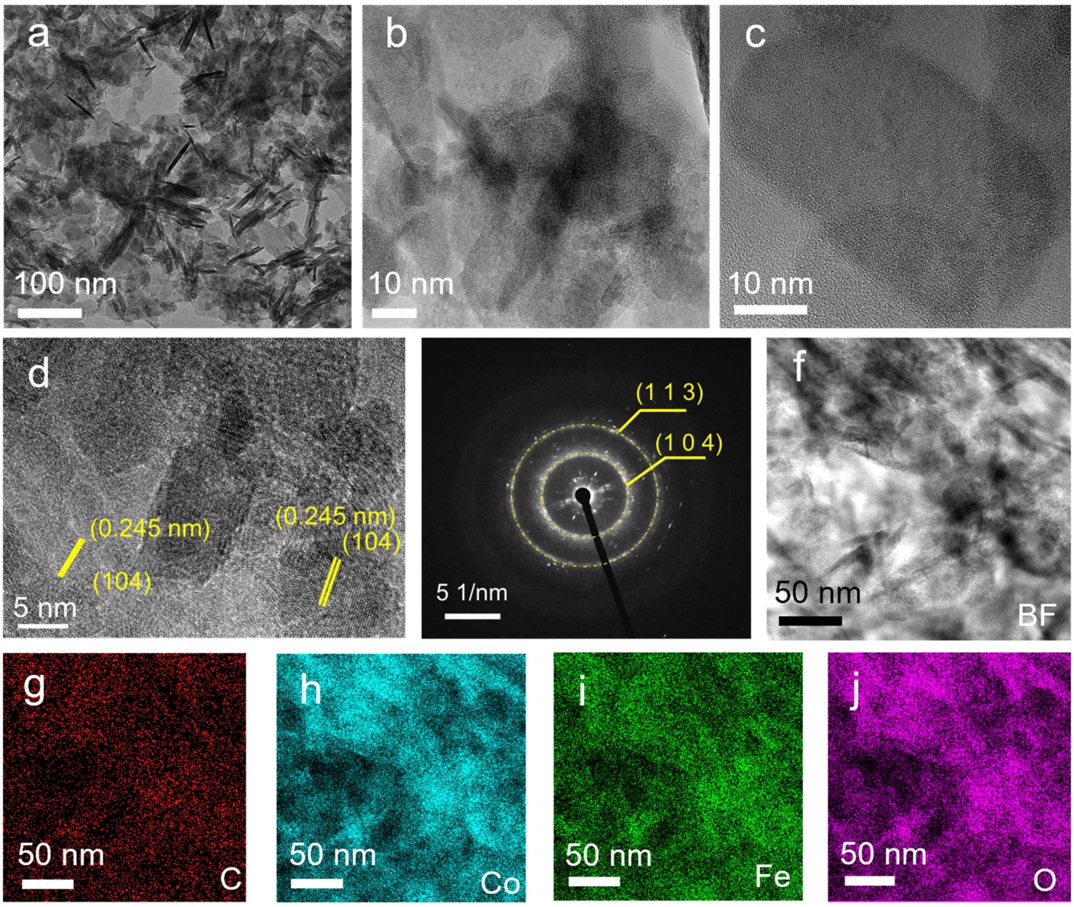

Fig. S6 Structural characterization of Co_4Fe_1. (**a-c**) TEM images of Co_4Fe_1 nanosheets at different magnifications; The (**d**) HRTEM image; (**e**) SAED patterns, (**f**) HAADF-STEM, and (**g–i**) associated elemental mapping images of Co_4Fe_1 nanosheets.

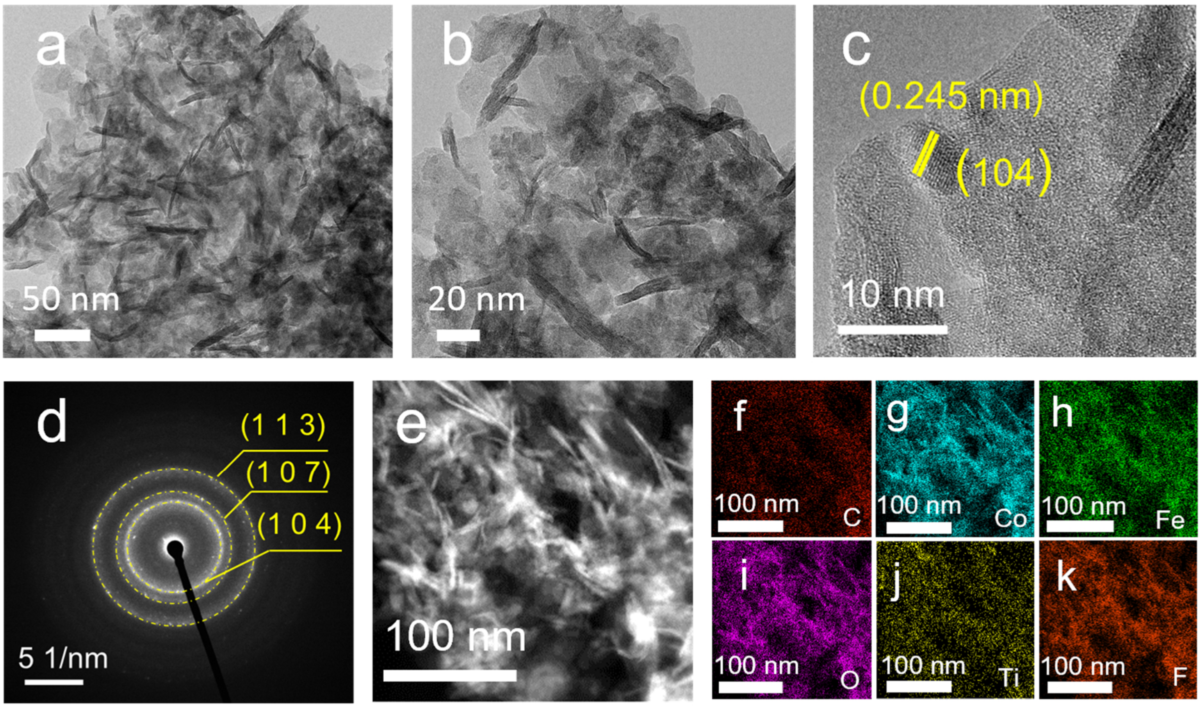

Fig. S7 Structural characterization of Co_3Fe_1-HO MX. (**a**,**b**) TEM images of Co_3Fe_1-HO MX. The (**c**) HRTEM image, (**d**) SAED patterns, (**e**) HAADF-STEM, and (**f**–**k**) associated elemental mapping images of Co_3Fe_1-HO MX nanosheets.

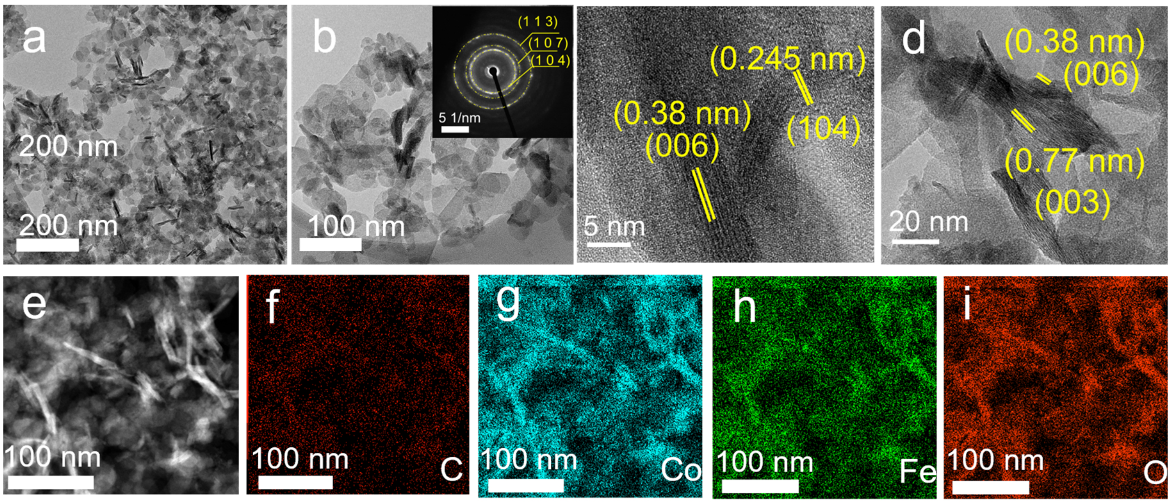

Fig. S8 Structural characterization of Co_3Fe_1. (**a,b**) TEM images of Co_3Fe_1, inset in **b** shows the corresponding SAED patterns. The (**c,d**) HRTEM image, (**e**) HAADF-STEM, and (**f–i**) associated elemental mapping images of Co_3Fe_1 nanosheets.

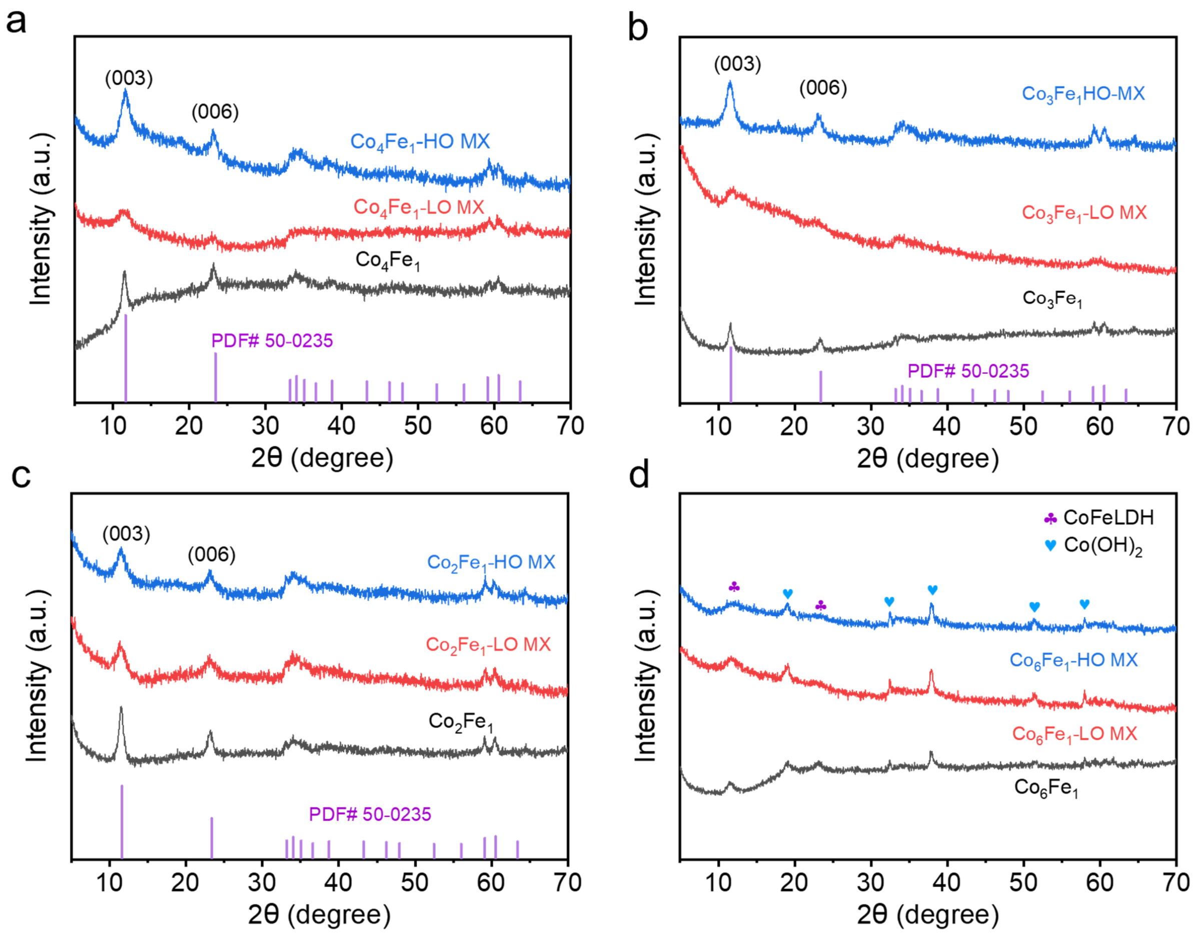

Fig. S9 XRD patterns of samples. XRD patterns of (**a**) pristine Co_4Fe_1, Co_4Fe_1-LO MX and Co_4Fe_1-HO MX; (**b**) pristine Co_4Fe_1, Co_3Fe_1-LO MX and Co_3Fe_1-HO MX; (**c**) pristine Co_2Fe_1, Co_2Fe_1-LO MX and Co_2Fe_1-HO MX, and (**d**) pristine Co_6Fe_1, Co_6Fe_1-LO MX and Co_6Fe_1-HO MX.

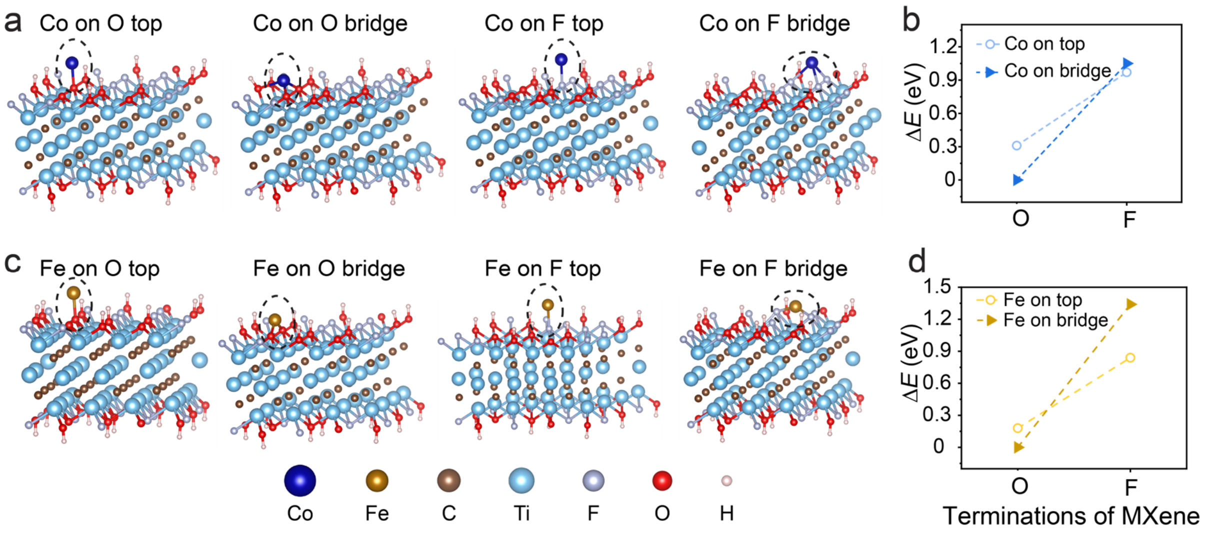

Fig. S10 DFT calculations for binding energy of Co and Fe absorb on MXene surface under vacuum. (**a**) View of the optimized structures for Co adsorption at different sites of O- and F- terminations on MXene ($F_{0.55}OH_{0.38}O_{0.19}$ (average of the HO and LO)) without using solvation correction. (**b**) The binding energy of Co at different adsorption sites on MXene, including O- and F- terminations. (**c**) views of the optimized structures for Fe adsorption at different sites of O- and F- terminations on MXene. (**d**) The binding energy of Fe at different adsorption sites on MXene, including O- and F- terminations.

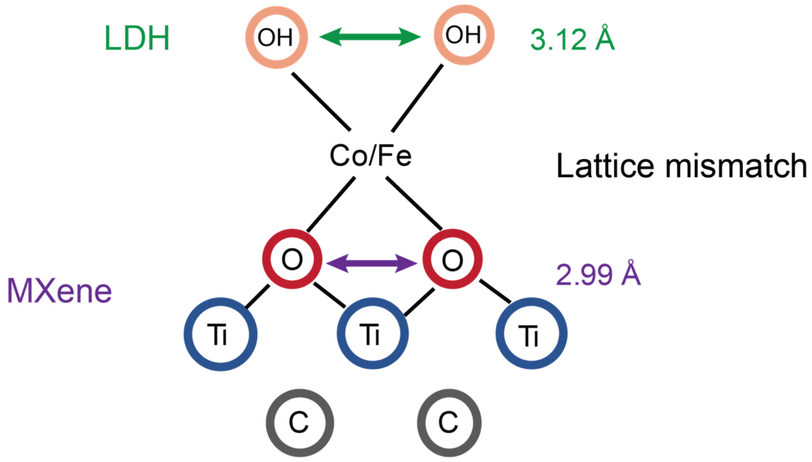

Fig. S11 Proposed mechanism of domain size decrease of CoFeLDH caused by lattice mismatch.

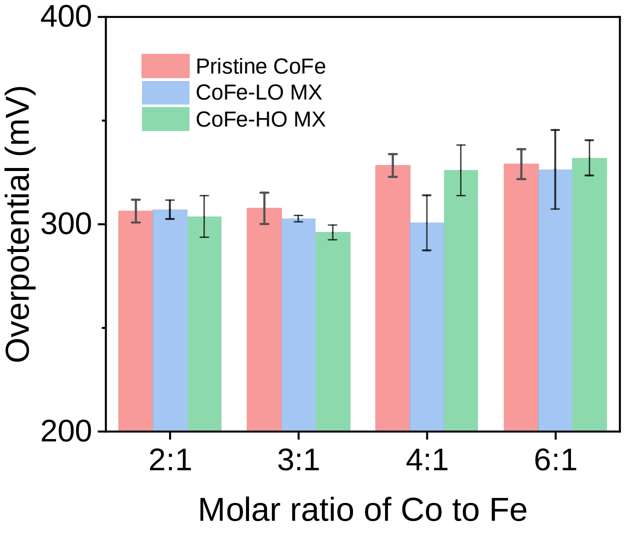

Fig. S12 iR-corrected average overpotential of as-prepared catalysts at 10 mA cm^{-2} at 5 mV/s scan rate. Error bars represent standard deviation obtained from 3 independent measurements.

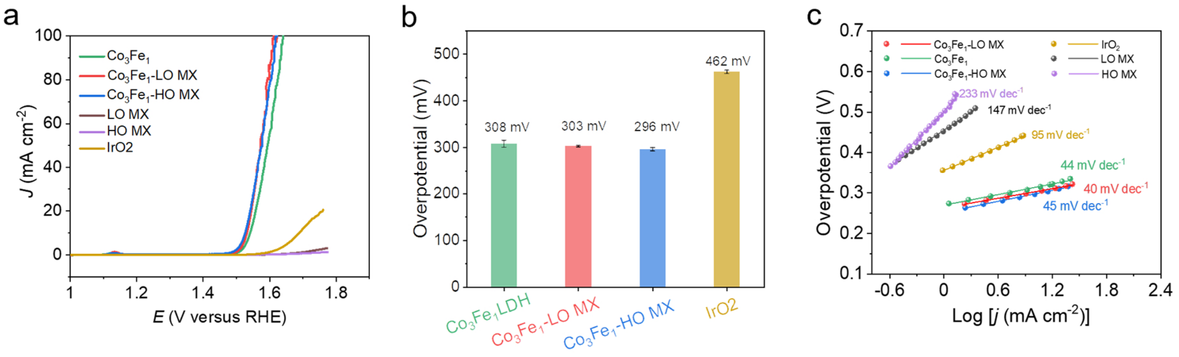

Fig. S13 OER performance for Co_3Fe_1 and related samples. (**a**) iR corrected-OER lsv curves, (**b**) overpotentials at 10 mA cm^{-2}, and (**c**) corresponding Tafel plots of Co_3Fe_1-HO MXene and other products, commercial IrO_2. Error bars represent standard deviation obtained from 3 independent measurements.

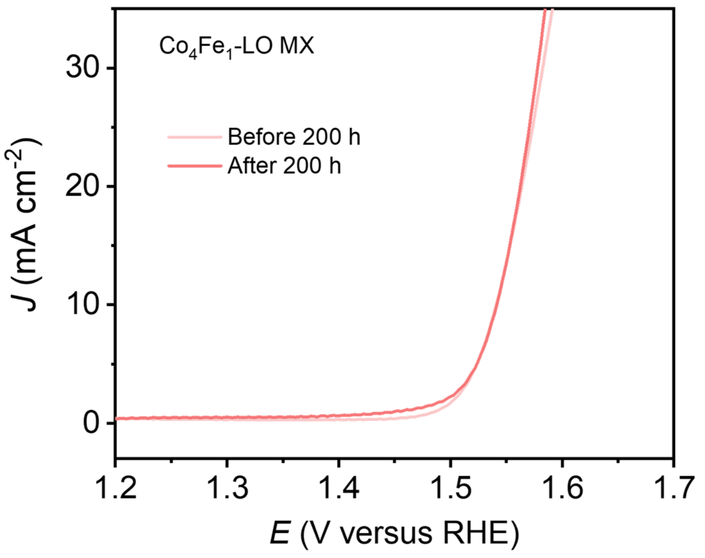

Fig. S14 LSV curves of Co_4Fe_1-LO MX before and after 200 h chronopotentiometry test.

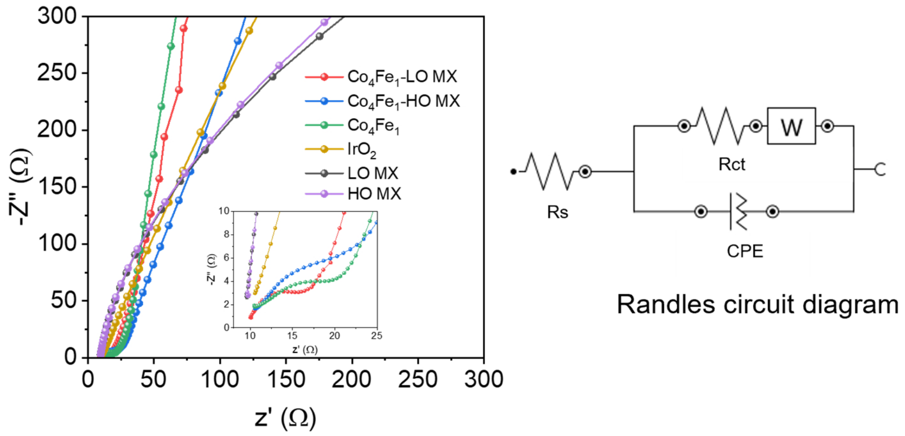

Fig. S15 EIS spectra of various catalysts toward OER.

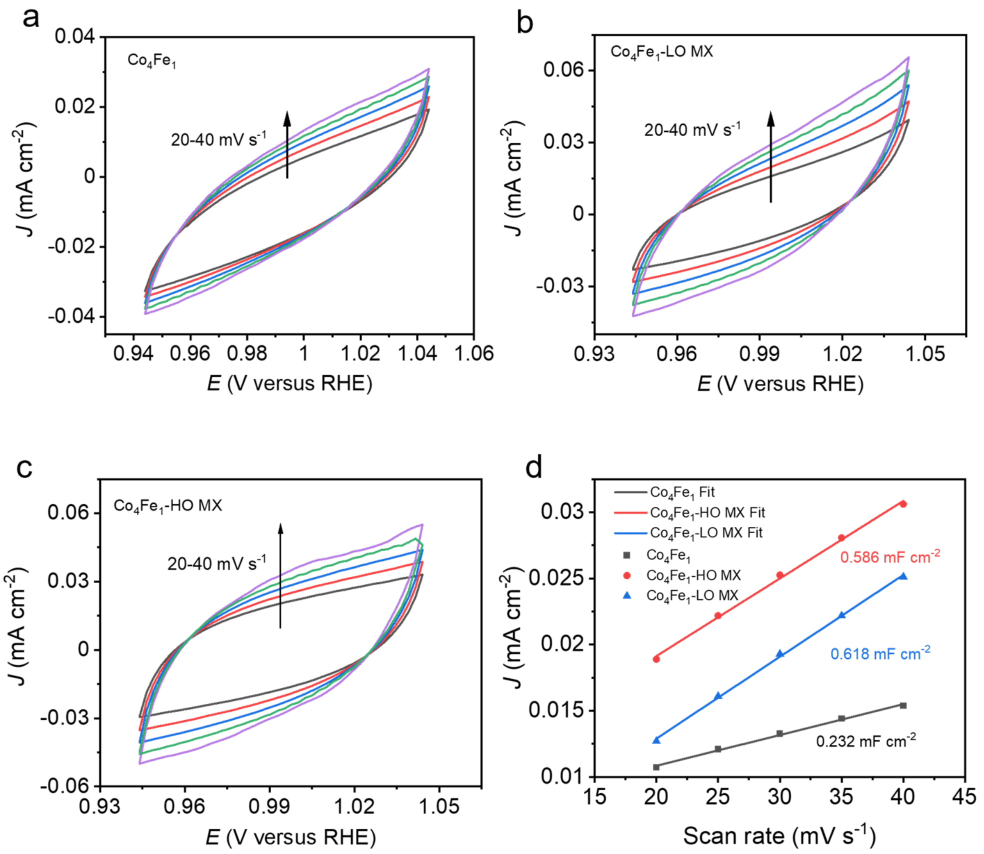

Fig. S16 CV curves and Cdl of Samples. CV curves of (**a**) Co_4Fe_1LDH, (**b**) Co_4Fe_1-LO MX, and (**c**) Co_4Fe_1-HO MX with different scan rates from 20 to 40 mV s^{-1}. (**d**) Plots of the Δj/2 against the scan rate at the potential of 0.994 V vs. RHE.

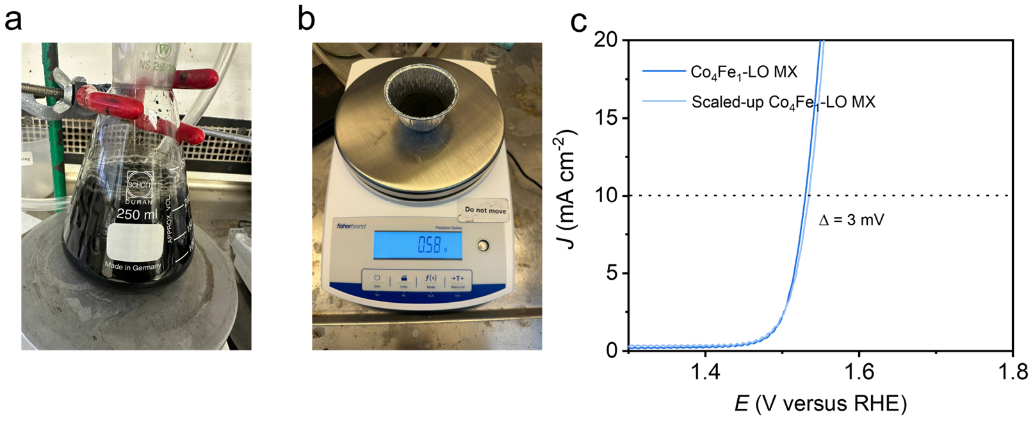

Fig. S17 Scaled-up synthesis of Co_4Fe_1-LO MX by 10 times. (a) Digital image of the synthesis process. (b) Digital image showing the product weight. (c) LSV comparison of small-scale and large-scale synthesized samples.

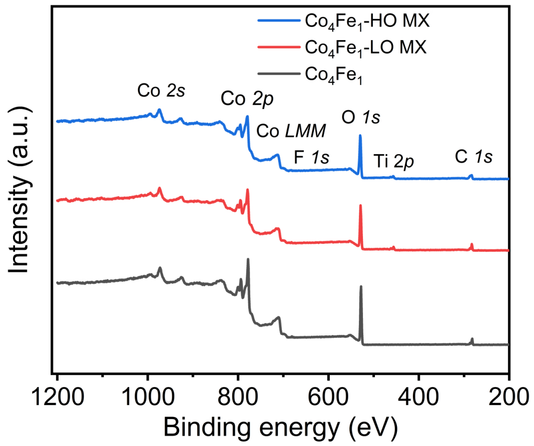

Fig. S18 XPS survey spectra of Co_4Fe_1, Co_4Fe_1-LO MX, and Co_4Fe_1-HO MX.

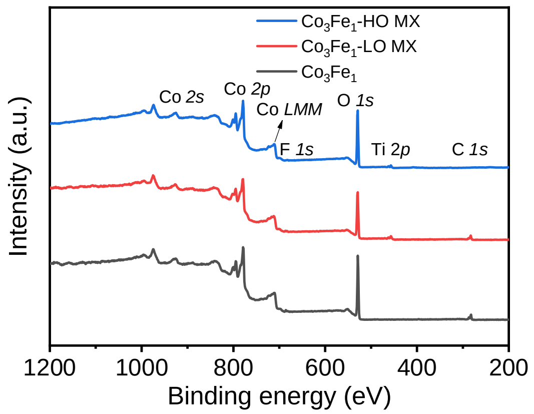

Fig. S19 XPS survey spectra of Co_3Fe_1, Co_3Fe_1-LO MX, and Co_3Fe_1-HO MX.

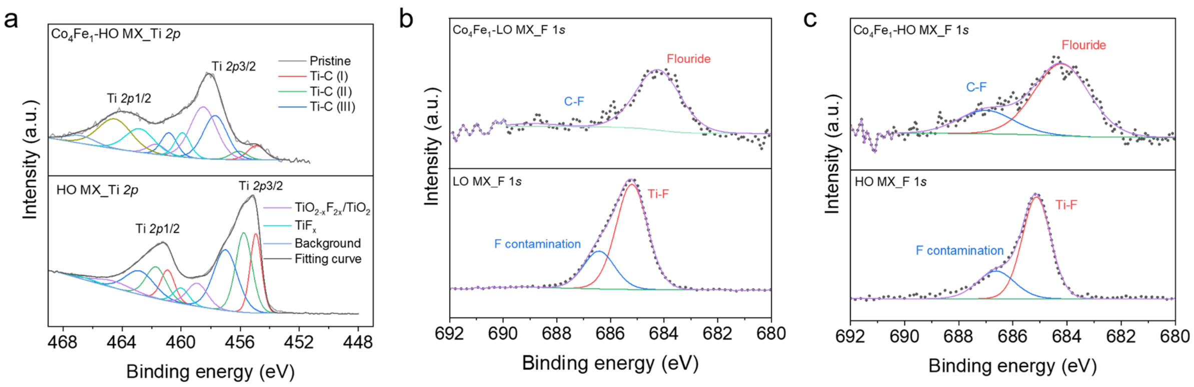

Fig. S20 Comparision of Ti 2p and F 1s spectra in Co₄Fe₁-MX samples. (**a**) Ti 2p spectra of HO MX and Co_4Fe_1-HO MX. F 1s spectra of (**b**) LO MX, Co_4Fe_1-LO MX, and (**c**) HO MX and Co_4Fe_1-HO MX.

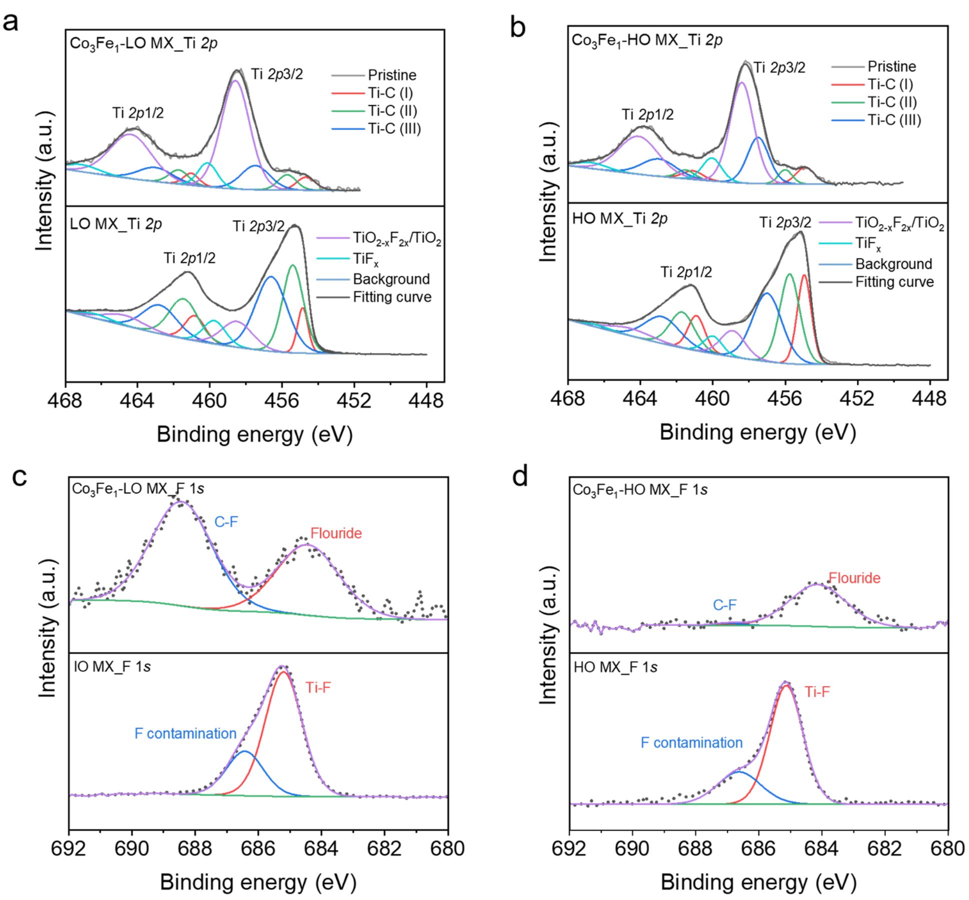

Fig. S21 Comparision of Ti 2p and F 1s spectra in Co_3Fe_1-MX samples. Ti 2p spectra of (**a**) LO MX and Co_3Fe_1-LO MX, and (**b**) HO MX, and Co_3Fe_1-HO MX. F 1s spectra of (**c**) LO MX and Co_3Fe_1-LO MX, and (**d**) HO MX, and Co_3Fe_1-HO MX.

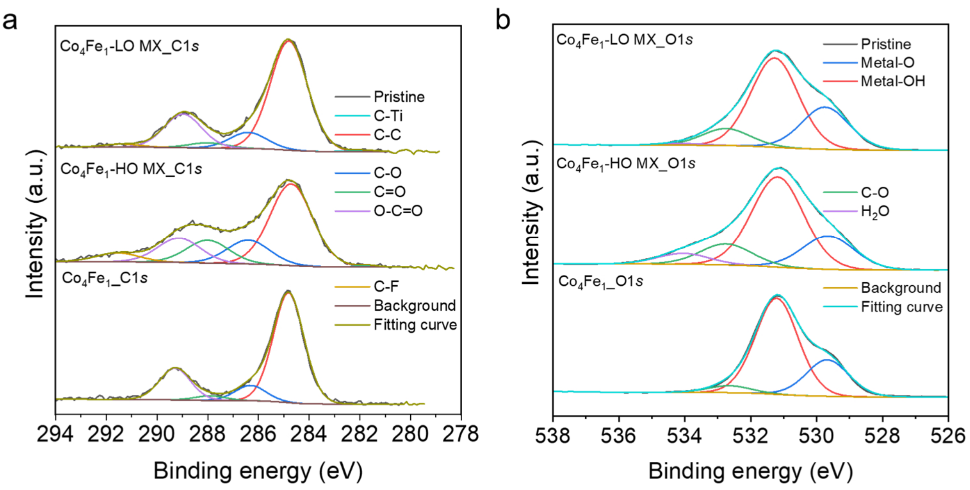

Fig. S22 Comparision of C 1s and O 1s spectra in Co_4Fe_1-MX samples. (**a**) C 1s spectra and (**b**) O 1s spectra of Co_4Fe_1, Co_4Fe_1-HO MX and Co_4Fe_1-HO MX.

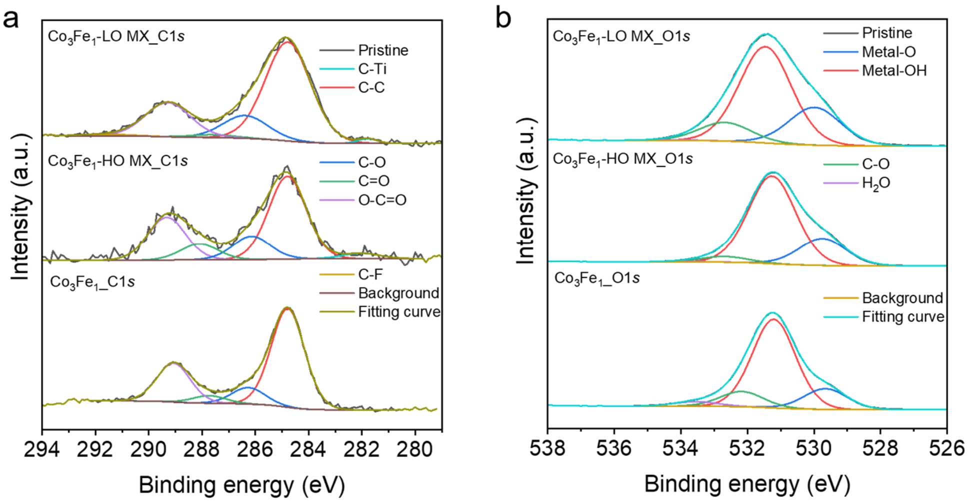

Fig. S23 Comparision of C 1s and O 1s spectra in Co_3Fe_1-MX samples. (**a**) C 1s spectra and (**b**) O 1s spectra of Co_3Fe_1, Co_3Fe_1-HO MX and Co_3Fe_1-HO MX.

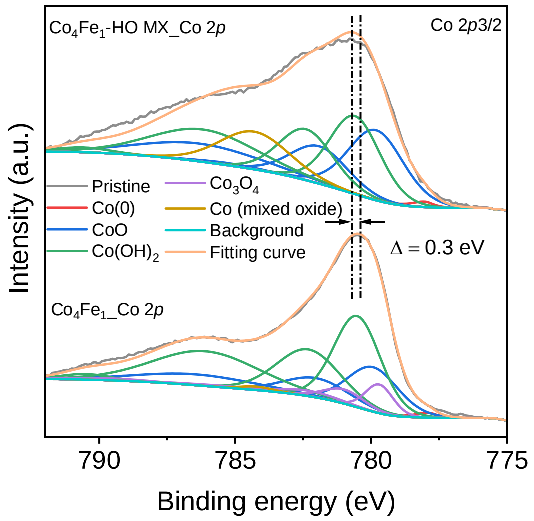

Fig. S24 Comparison of Co 2p spectra in pristine Co_4Fe_1 and Co_4Fe_1-HO MX.

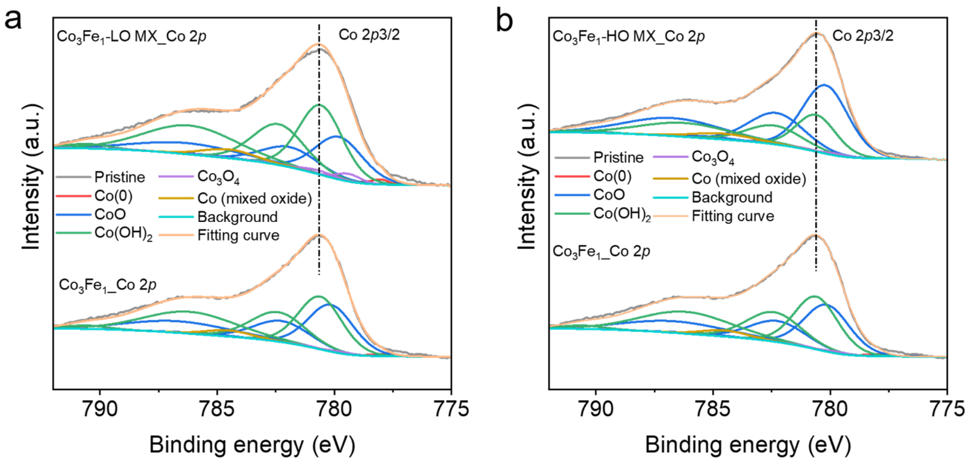

Fig. S25 Comparison of Co 2p spectra in Co_3Fe_1-MX samples. (**a**) pristine Co_3Fe_1 and Co_3Fe_1-LO MX, and (**b**) pristine Co_3Fe_1 and Co_3Fe_1-HO MX.

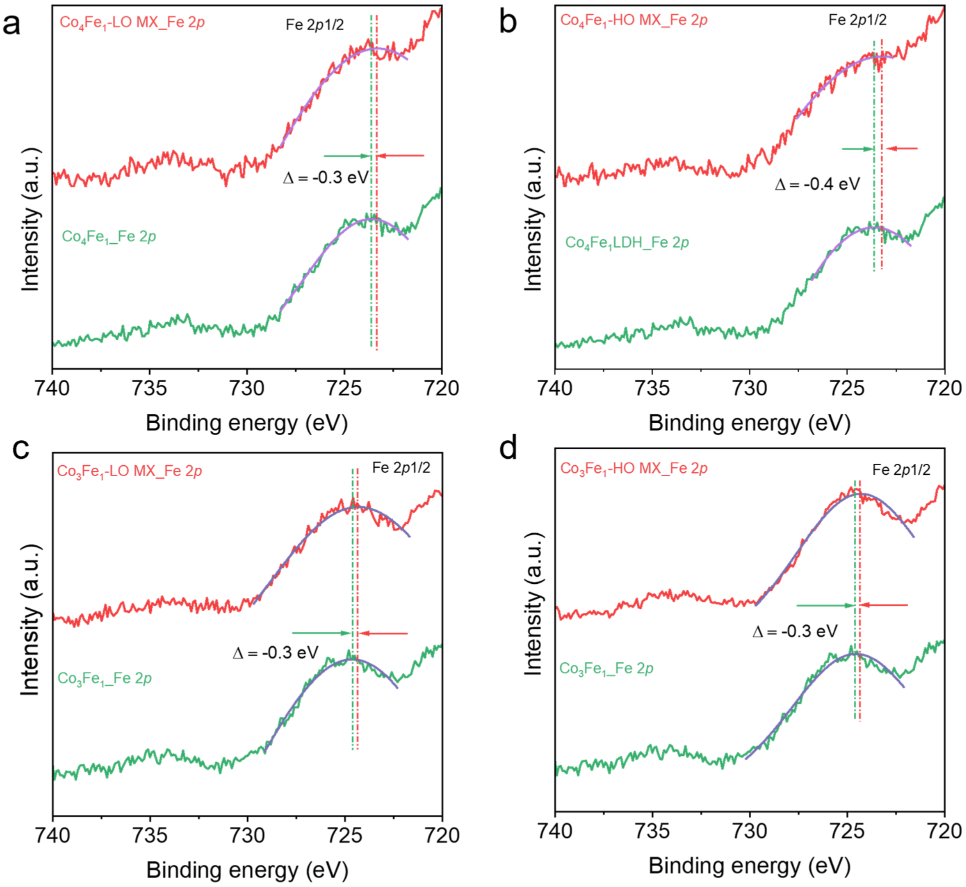

Fig. S26 Comparison of Fe 2p spectra of samples. Fe 2p for (**a**) pristine Co_4Fe_1 and Co_4Fe_1-LO MX, (**b**) pristine Co_4Fe_1LDH and Co_4Fe_1-HO MX, (**c**) pristine Co_3Fe_1 and Co_3Fe_1-LO MX, and (**d**) pristine Co_3Fe_1 and Co_3Fe_1-HO MX.

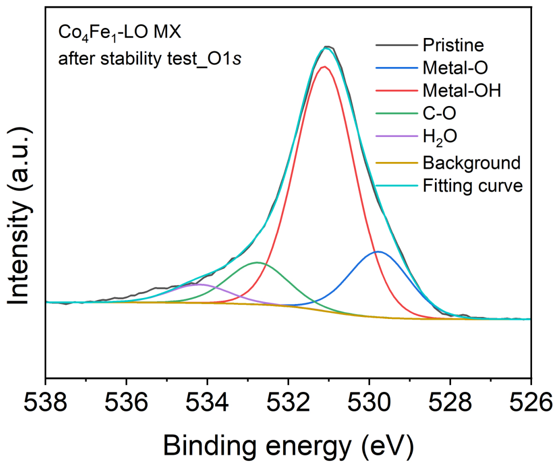

Fig. S27 XPS spectrum of Co_4Fe_1-LO MX for O 1s after stability test.

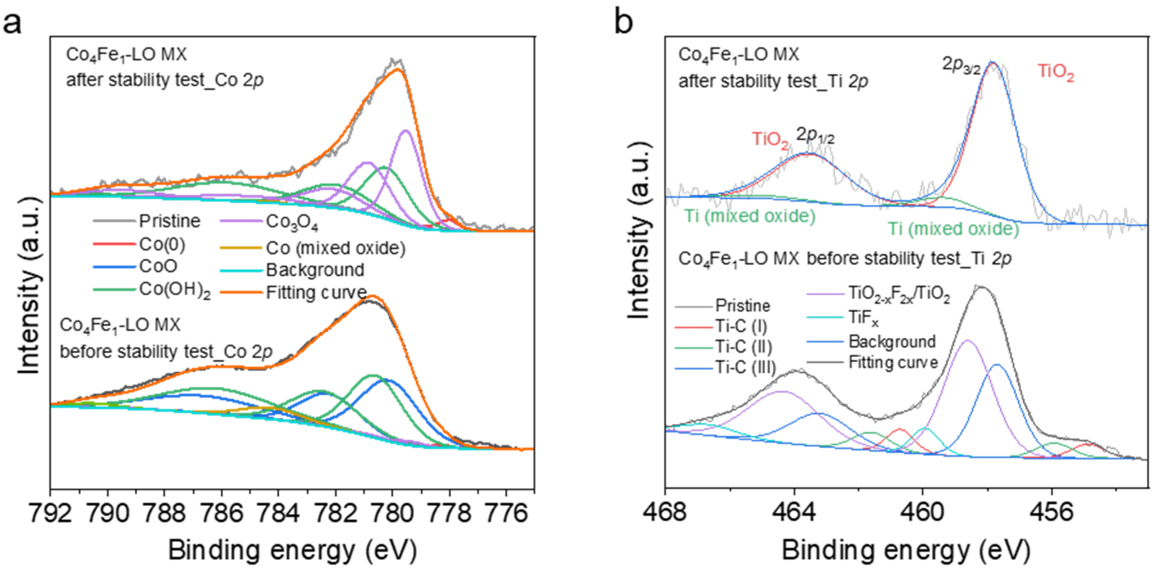

Fig. S28 Post-catalytic XPS characterization of Co_4Fe_1-LO MX. (**a**) XPS spectra of Co 2p, and (**b**) Ti 2p of Co_4Fe_1-LO MX after stability test.

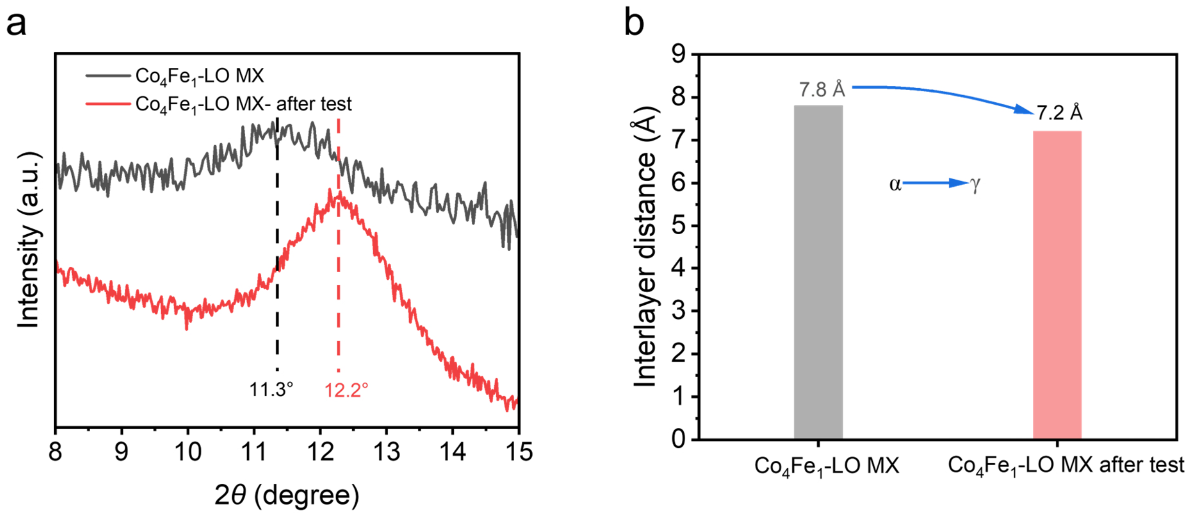

Fig. S29 Post-catalytic XRD characterization of Co_4Fe_1-LO MX.. (a) XRD patterns, and (b) interlayer distances of Co_4Fe_1-LO MX before and after stability test

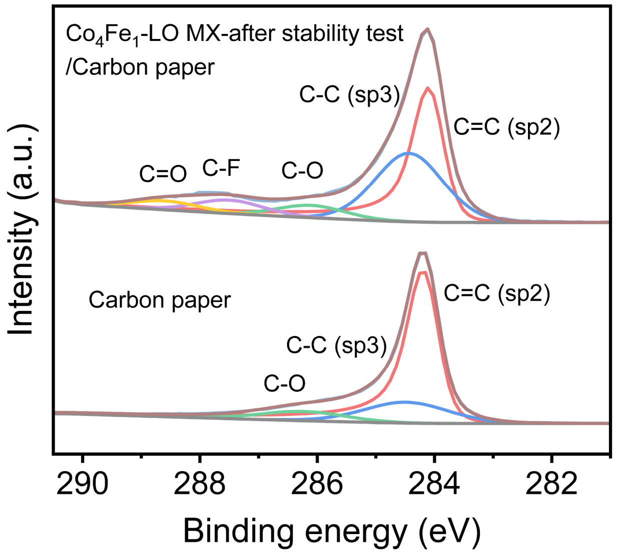

Fig. S30 Post-Catalytic XPS Characterization of Co_4Fe_1-LO MX with C 1s Spectra Analysis.

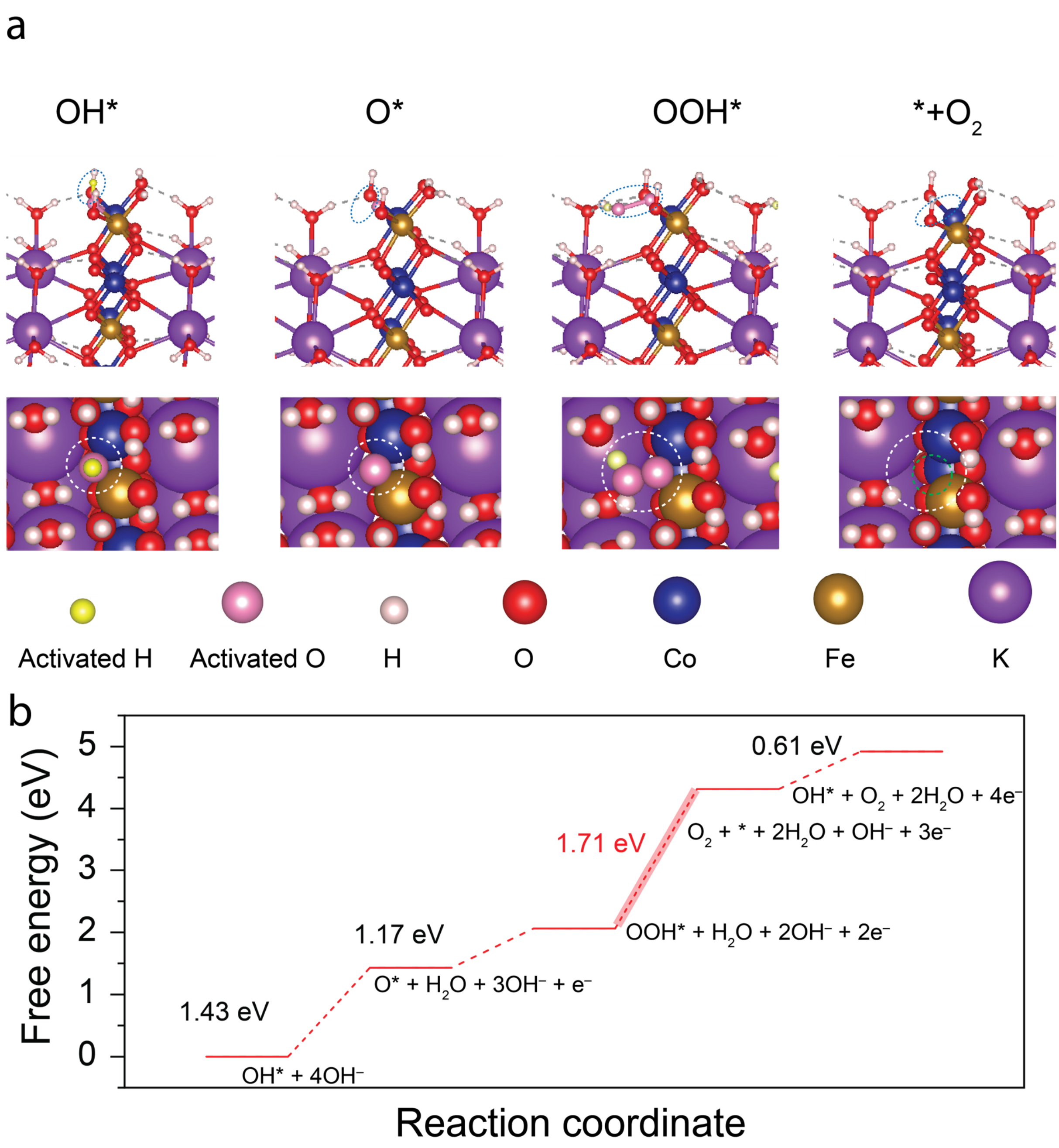

Fig. S31 Free energy profiles of pristine γ-CoFeLDH (model 1) for OER (**a**) Different surface phase structures and OER intermediates of pristine γ-Co_3Fe_1LDH are illustrated with blue circles denoting adsorbates on the side views. OER intermediates are color-coded for clarity, with yellow representing hydrogen and rose indicating oxygen (in place of white and red, respectively). A dashed rose circle signifies the formation of a surface O vacancy. Reaction centers are highlighted by large white circles. (**b**) Reaction free-energy diagrams for OER on the pristine CoFeLDH with highlighting potential limiting steps.

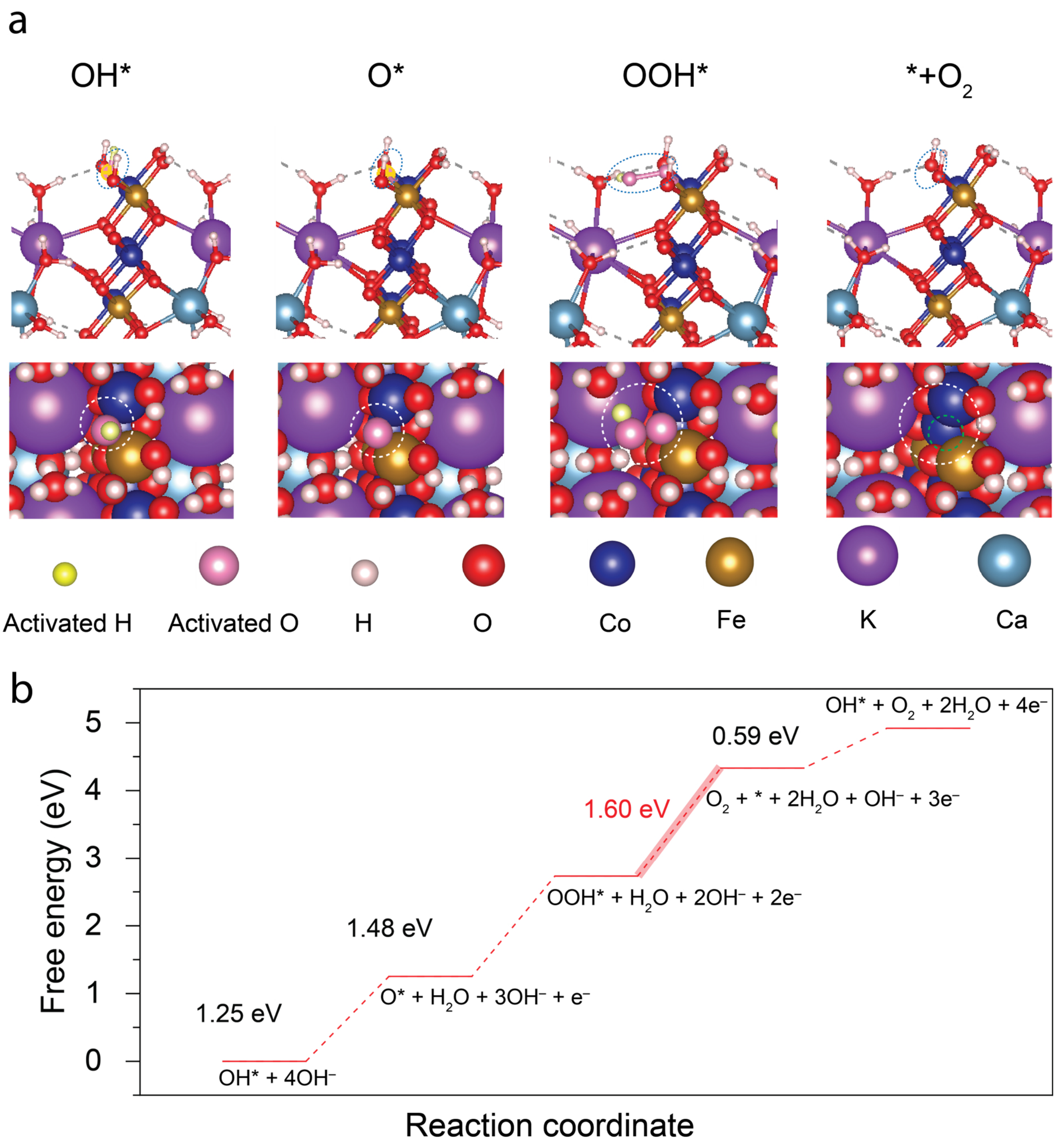

Fig. S32 Free energy profiles of CoFeLDH-$Ti_3C_2T_x$ (model 2) for OER (**a**) Different surface phase structures and OER intermediates of pristine γ-Co_3Fe_1LDH (K by Ca in model 2) are illustrated with blue circles denoting adsorbates on the side views. OER intermediates are color-coded for clarity, with yellow representing hydrogen and rose indicating oxygen (in place of white and red, respectively). A dashed green circle signifies the formation of a surface O vacancy. Reaction centers are highlighted by large white circles. (**b**) Reaction free-energy diagrams for OER on the pristine γ-Co_3Fe_1LDH with highlighting potential limiting steps.

Supporting Tables

Table S1 XPS analysis of LO MX.

Region	BE (eV)	FWHM (eV)	Fraction (%)	Assigned to	Reference
Ti $2p_{3/2}$ ($2p_{1/2}$)	454.9 (460.8)	0.7 (1.3)	0.83	C-Ti (I)	[17]
	455.4 (461.5)	1.4 (1.8)	2.54	C-Ti (II)	[17]
	456.6 (462.8)	2.1 (2.2)	2.85	C-Ti (III)	[17]
	458.5 (464.8)	2.0 (2.6)	1.02	$TiO_{2-x}F_x/TiO_2$	[17]
	459.8 (466.3)	1.4 (2.0)	0.56	TiF_x	[17]
C 1s	281.9	0.6	4.50	Ti-C-Ti	[17]
	282.6	1.6	2.05	C-Ti	[17]
	284.8	1.2	4.38	C-C	[17]
	285.9	1.4	55.57	C-O	[17]
	287.9	1.9	1.88	C=O	[17]
	289.7	1.4	3.22	O-C=O	[17]
O 1s	529.8	0.8	1.16	C-Ti-O (i)	[17]
	530.0	1.8	1.24	$TiO_{2-x}F_{2x}$	[17]
	531.1	1.5	1.56	C-Ti-O (ii)	[17]
	532.3	1.4	1.33	C-Ti-OH	[17]
	532.8	1.6	7.18	OR	[17]
	534.1	2.9	1.43	H_2O	[17]
F 1s	685.2	1.4	4.94	Ti-F	[17]
	686.4	1.4	1.76	F contamination	[17,18]

Table S2 XPS analysis of HO MX.

Region	BE (eV)	FWHM (eV)	Fraction (%)	Assigned to	Reference
Ti $2p_{3/2}$ ($2p_{1/2}$)	454.9 (460.9)	0.9 (1.1)	1.14	C-Ti (I)	[17]
	455.7 (461.7)	1.3 (1.6)	1.67	C-Ti (II)	[17]
	457.0 (462.8)	1.9 (2.3)	1.84	C-Ti (III)	[17]
	458.9 (464.6)	1.6 (2.9)	0.61	$TiO_{2-x}F_x$/TiO_2	[17]
	459.8 (466.3)	1.2 (2.0)	0.25	TiF_x	[17]
C 1s	281.9	0.6	3.29	Ti-C-Ti	[17]
	282.6	1.7	1.65	C-Ti	[17]
	284.8	1.3	3.34	C-C	[17]
	285.9	1.4	67.47	C-O	[17]
	287.7	1.7	1.18	C=O	[17]
	289.7	1.4	3.88	O-C=O	[17]
O 1s	529.7	0.9	1.30	C-Ti-O (i)	[17]
	530.5	1.1	0.45	$TiO_{2-x}F_{2x}$	[17]
	531.1	1.5	0.29	C-Ti-O (ii)	[17]
	532.1	1.3	2.18	C-Ti-OH	[17]
	532.9	1.4	6.12	OR	[17]
	533.8	2.3	0.97	H_2O	[17]
F 1s	685.1	1.2	1.77	Ti-F	[17]
	686.6	1.6	0.63	F contamination	[17,18]

Table S3 Atomic concentrations (atomic-%) and the O to F ratios in HO MX and LO MX. According to the table above, after substrate the TiO_2 of Ti 2p; C-O, C=O and O-C=O of C 1s, probably come from organic contamination; TiO_2, OR and H_2O of O 1s; F contamination of F1s, we can correct the proportions of each element in the following table.

Sample	C %	O %	Ti %	F %	O : F	Ti:(F+O)
HO MX	44.2	20.1	26.2	9.5	2.12 : 1	0.89 : 1
LO MX	40.9	15.2	25.4	18.5	0.82 : 1	0.75 : 1

Table S4 Calculated *d*-spacing from each lamellar peak in HO MX and LO MX. The standard deviation (SD) of *d* from all peaks is also given.

Sample	HO MX			LO MX		
Peaks	2 theta (°)	*d* (Å)	SD (Å)	2 theta (°)	*d* (Å)	SD (Å)
(002)	5.986	14.75		5.99	14.75	
(004)	12.01	14.72		12.02	14.72	
(006)	18.00	14.77		18.09	14.70	
(008)	24.14	14.74	0.017	24.38	14.59	0.05
(0010)	30.24	14.77		30.39	14.69	
(0012)	36.51	14.75		36.71	14.68	
(0014)	42.88	14.75		43.07	14.69	

Table S5 The atomic ratio of Co/Fe for as-prepared samples rely on the ICP-OES analysis.

**Samples**	**Feed ratio of Co/Fe**	**Ratio of Co/Fe determined by ICP-OES**
Co_4Fe_1	4/1	4.31/1
Co_4Fe_1-LO MX	4/1	4.26/1
Co_4Fe_1-HO MX	4/1	4.31/1
Co_3Fe_1	3/1	3.57/1
Co_3Fe_1-LO MX	3/1	3.34/1
Co_3Fe_1-HO MX	3/1	3.28/1
Co_2Fe_1	2/1	2.14/1
Co_2Fe_1-LO MX	2/1	2.12/1
Co_2Fe_1-HO MX	2/1	2.21/1
Co_6Fe_1	6/1	6.38/1
Co_6Fe_1-LO MX	6/1	6.08/1
Co_6Fe_1-HO MX	6/1	6.06/1

Table S6 Domain size calculated from XRD results.

**Samples**	**Peak position(2theta)**	**FWHM (β)**	**Size (nm)**
Co_4Fe_1	11.51	0.6314	12.64
Co_4Fe_1-LO MX	11.29	2.152	3.71
Co_4Fe_1-HO MX	11.66	1.320	6.05
Co_3Fe_1	11.57	0.6544	12.20
Co_3Fe_1-LO MX	12.04	2.431	3.28
Co_3Fe_1-HO MX	11.51	1.325	6.02
Co_2Fe_1	11.53	0.7749	10.30
Co_2Fe_1-LO MX	11.38	1.388	5.75
Co_2Fe_1-HO MX	11.47	1.336	5.97
Co_6Fe_1 (LDH (003))	11.62	0.9540	8.37
Co_6Fe_1-LO MX (LDH (003))	11.70	1.795	4.45
Co_6Fe_1-HO MX (LDH (003))	11.95	2.060	3.88
Co_6Fe_1 ($Co(OH)_2$ (001))	19.13	0.9777	8.24
Co_6Fe_1-LO MX ($Co(OH)_2$ (001))	18.93	0.9009	8.94
Co_6Fe_1-HO MX ($Co(OH)_2$ (001))	18.96	0.9102	8.85

Table S7 Overpotential, Tafel slope and stability at 10 mA cm^{-2} of Co_4Fe_1LDH-LO MX and previously reported MXene heterostructures.

Materials	Overpotential (mV)	Tafel slope (mV dec^{-1})	Stability (h)	References
NiFeLDH/Ti_3C_2-MXene	298	43	12	[19]
CoP@3D MXene	298	51	10	[20]
CoFeLDH/MXene	319	50	10	[21]
CoFeLDH nanosheet	314	79.4	12	[22]
CoFeLDH-F	300	95	12.5	[23]
g-C_3N_4/MXene	330	74.6	10	[24]
Co-LDH@Ti_3C_2Tx	330	82	20	[25]
NiCoS/$Ti_3C_2T_x$	365	58.2	5.6	[26]
BP QDs/MXene	360	64.3	10	[27]
$MoSe_2$/MXene	340	90	50	[28]
NiFeLDH	348	--	--	[10]
NiFeLDH	348	--	--	[9]
NiFe-LDH/Fe1-N-C	320	41	--	[29]
Co_4Fe_1-LO MX	301	43	200 (0.1% decay)	This work

Table S8 Relative concentrations of the different components of cobalt. These are deconvoluted from the Co 2p3/2 peak in the samples as compared to the total amount of cobalt in the samples.

Sample	Co (0) %	CoO %	$Co(OH)_2$ %	Co (mixed oxide) %	Co_3O_4 %
Co_4Fe_1	0.38	30.23	59.37	1.03	8.98
Co_4Fe_1-LO MX	0.16	44.12	50.49	2.62	2.62
Co_4Fe_1-HO MX	0.73	40.24	48.98	9.91	0.15
Co_4Fe_1-LO MX (after stability test)	2.27	0.00	29.62	0.08	68.03
Co_3Fe_1	0.35	41.32	54.14	3.70	0.50
Co_3Fe_1-LO MX	1.19	30.21	62.72	3.00	2.88
Co_3Fe_1-HO MX	0.12	60.77	34.8	3.2	1.11

Table S9 DFT calculated free energy contributions for all configurations of pristine CoFeLDH.

	OH*	O*	OOH*	*
Energy (eV)	−200.4257216	−195.3475484	−203.3588229	−191.9316071
ZPE (eV)	0.3832	0.0810	0.4613	
TS (eV)	0.0571	0.0369	0.1091	
Delta H (eV)	0.0367	0.0243	0.0646	
E solvation (eV)	−0.5483	−0.4742	−0.4270	−0.4971

Table S10 Reaction Gibbs free energy of pristine CoFeLDH.

Reaction	δG (eV)	δδG
OH* → O*	1.43	1.43
OH* → OOH*	2.60	1.17
OH* → V + O_2	4.31	1.71
OH* → OH* + O_2	4.92	0.61

Table S11 DFT calculated free energy contributions for all configurations of CoFeLDH-$Ti_3C_2T_x$ model 1 with one added electron.

	OH*	O*	OOH*	*
Energy (eV)	−202.1775039	−197.2262442	−205.0031947	−193.7456239
ZPE (eV)	0.3780	0.0805	0.4626	
TS (eV)	0.0606	0.0371	0.1130	
Delta H (eV)	0.0385	0.0245	0.0660	
E solvation (eV)	−0.5889	−0.5655	−0.4901	−0.5609

Table S12 Reaction Gibbs free energy of model 1.

	δG (eV)	δδG (eV)
OH* → O*	1.26	1.26
OH* → OOH*	2.69	1.43
OH* → V + O_2	4.24	1.55
OH* → OH* + O_2	4.92	0.68

Table S13 DFT calculated free energy contributions for all configurations of CoFeLDH-$Ti_3C_2T_x$ model 2 with one K replaced by Ca.

	OH*	O*	OOH*	*
Energy (eV)	−204.9243568	−199.9655912	−207.7041967	−196.4276478
ZPE (eV)	0.3897	0.0801	0.4633	
TS (eV)	0.0536	0.0375	0.1103	
Delta H (eV)	0.0348	0.0247	0.0655	
E solvation (eV)	−0.5623	−0.5381	−0.4566	−0.4883

Table S14 Reaction Gibbs free energy of model 2

	δG (eV)	δδG (eV)
OH* → O*	1.25	1.25
OH* → OOH*	2.73	1.48
OH* → V + O_2	4.33	1.60
OH* → OH* + O_2	4.92	0.59

Table S15 Bader charge of active atoms for pristine CoFeLDH.

Atoms	OH*	O*	OOH*	*
Co	7.510	7.377	7.403	7.532
Fe	5.997	6.027	6.110	6.106
Bridge O	7.509	6.812	6.494	--

Table S16 Bader charge of active atoms for CoFeLDH-$Ti_3C_2T_x$ model 1.

Atoms	OH*	O*	OOH*	*
Co	7.523	7.395	7.411	7.550
Fe	6.009	6.114	6.122	6.131
Bridge O	7.563	6.839	6.529	--

Table S17 Bader charge of active atoms for CoFeLDH-$Ti_3C_2T_x$ model 2.

Atoms	OH*	O*	OOH*	*
Co	7.514	7.385	7.403	7.531
Fe	5.981	5.988	6.106	6.111
Bridge O	7.476	6.784	6.494	--

Table S18 DFT calculated magnetic moments of active atoms for pristine CoFeLDH.

Atoms	Magnetic moments (μB)			
	OH*	O*	OOH*	*
Co	0.024	−1.002	1.124	1.773
Fe	−3.430	−3.238	−1.944	−3.292
Bridge O	−0.048	−0.162	−0.036	--

Table S19 DFT calculated magnetic moments of active atoms for CoFeLDH-$Ti_3C_2T_x$ model 1.

Atoms	Magnetic moments (μB)			
	OH*	O*	OOH*	*
Co	0.031	−0.949	−1.041	1.769
Fe	−3.483	−1.883	−1.964	−3.282
Bridge O	−0.089	−0.122	0.060	--

Table S20 DFT calculated magnetic moments of active atoms for CoFeLDH-$Ti_3C_2T_x$ model 2.

Atoms	Magnetic moments (μB)			
	OH*	O*	OOH*	*
Co	−0.019	0.965	1.093	1.775
Fe	−2.723	−2.598	−1.992	−3.309
Bridge O	0.002	-0.155	-0.033	--

Table S21 Numerical values for free energy contributions to H_2 and H_2O molecules. Energy and ZPE are from DFT, while TS and Delta H are taken from NIST-JANAF thermochemical tables. The H_2O TS is obtained from the gas phase TS (0.5835 eV) and Gibbs free energy difference between gas and liquid phases (0.0887 eV).

	H_2	H_2O
Energy (eV)	−6.81685819	−12.21128846
ZPE (eV)	0.2713	0.5650
TS (eV)	0.4038	0.6722
Delta H (eV)	0.0930	0.1026

Table S22 The detailed comparison table of the highlights (excluding catalytic performance and mechanism) of this work with other similar works (“Y” stands for Yes, and “--" stands for No).

Materials	Charge transfer discussion		Regulated charge transfer	Surface analysis of MXene combining experimental and theoretical approaches		Termination guide growth mechanism	Catalyst reconstruction analysis	Scalability	Ref.
	Experimental	Theoretical		Experimental analysis	Theoretical analysis				
Co_4Fe_1-LO MX	Y	Y	Y	Y	Y	Y	Y	Y	This work
NiFeLDH/Ti_3C_2-MXene	Y	Y	--	--	--	--	--	--	[19]
CoP@3D MXene	Y		--	--	--	--	--	--	[20]
CoFeLDH/MXene	Y	Y	--	--	--	--	--	--	[21]
g-C_3N_4/MXene	Y		--	--	--	--	--	--	[24]
Co-LDH@Ti_3C_2Tx	Y		--	--	--	--	--	--	[25]
NiCoS/$Ti_3C_2T_x$	Y	--	--	--	--	--	--	--	[26]
BP QDs/MXene	--	Y	--	--	--	--	--	--	[27]
$MoSe_2$/MXene	Y	--	--	--	--	--	--	--	[28]
Fe/MXene	Y	--	--	--	--	--	Y	Y	[30]
Fe–N–C/MXene	Y	--	--	--	--	--	--	--	[31]

Reference

[1] U. Holzwarth, N. Gibson, *Nat. Nanotech.* **2011**, *6*, 534.
[2] Y. Duan, Z. Yu, S. Hu, X. Zheng, C. Zhang, H. Ding, B. Hu, Q. Fu, Z. Yu, X. Zheng, *Angew. Chem., Int. Ed.* **2019**, *58*, 15772.
[3] M. Gao, W. Sheng, Z. Zhuang, Q. Fang, S. Gu, J. Jiang, Y. Yan, *J. Am. Chem. Soc.* **2014**, *136*, 7077.
[4] J. Zhang, F. Xing, H. Zhang, Y. Huang, *Dalton Trans.* **2020**, *49*, 13962.
[5] R. Ibragimova, M. J. Puska, H.-P. Komsa, *ACS nano* **2019**, *13*, 9171.
[6] A. V. D. Walle, *Calphad* **2009**, *33*, 266.
[7] A. van de Walle, G. Ceder, *J. Phase Equilib.* **2002**, *23*, 348.
[8] A. V. D. Walle, M. Asta, *Modell. Simul. Mater. Sci. Eng.* **2002**, *10*, 521.
[9] F. Dionigi, J. Zhu, Z. Zeng, T. Merzdorf, H. Sarodnik, M. Gliech, L. Pan, W. X. Li, J. Greeley, P. Strasser, *Angew. Chem., Int. Ed.* **2021**, *60*, 14446.
[10] F. Dionigi, Z. Zeng, I. Sinev, T. Merzdorf, S. Deshpande, M. B. Lopez, S. Kunze, I. Zegkinoglou, H. Sarodnik, D. Fan, *Nat. Commun.* **2020**, *11*, 1.
[11] J. Klimeš, D. R. Bowler, A. Michaelides, *Journal of Physics: Condens. Matter* **2009**, *22*, 022201.
[12] G. Kresse, J. Furthmüller, *Comput. Mater. Sci.* **1996**, *6*, 15.
[13] G. Kresse, J. Furthmüller, *Phys. Rev. B* **1996**, *54*, 11169.
[14] G. Kresse, D. Joubert, *Phys. Rev. B* **1999**, *59*, 1758.
[15] K. Mathew, R. Sundararaman, K. Letchworth-Weaver, T. A. Arias, R. G. Hennig, *J. Chem. Phys.* **2014**, *140*, 084106.
[16] J. M. W. Chase, *NIST-JANAF Thermochemical Tables*, Fourth Edition. Washington, DC : American Chemical Society ; New York : American Institute Of Physics For The National Institute Of Standards And Technology, 1998., **1998**.
[17] J. Halim, K. M. Cook, M. Naguib, P. Eklund, Y. Gogotsi, J. Rosen, M. W. Barsoum, *Appl. Surf. Sci.* **2016**, *362*, 406.
[18] A. Tanvir, P. Sobolčiak, A. Popelka, M. Mrlik, Z. Spitalsky, M. Micusik, J. Prokes, I. Krupa, *Polymers* **2019**, *11*, 1272.
[19] M. Yu, S. Zhou, Z. Wang, J. Zhao, J. Qiu, *Nano Energy* **2018**, *44*, 181.
[20] L. Xiu, Z. Wang, M. Yu, X. Wu, J. Qiu, *ACS Nano* **2018**, *12*, 8017.
[21] C. Hao, Y. Wu, Y. An, B. Cui, J. Lin, X. Li, D. Wang, M. Jiang, Z. Cheng, S. Hu, *Mater. Today Energy* **2019**, *12*, 453.
[22] Y. Zhang, C. Gan, Q. Jiang, P. Lang, W. Wang, J. Tang, *Mater. Sci. Eng. B.* **2022**, *282*, 115800.
[23] P. F. Liu, S. Yang, B. Zhang, H. G. Yang, *ACS Appl. Mater. Interfaces* **2016**, *8*, 34474.
[24] T. Y. Ma, J. L. Cao, M. Jaroniec, S. Z. Qiao, *Angew. Chem., Int. Ed.* **2016**, *55*, 1138.
[25] M. Benchakar, T. Bilyk, C. Garnero, L. Loupias, C. Morais, J. Pacaud, C. Canaff, P. Chartier, S. Morisset, N. Guignard, *Adv. Mater. Interfaces* **2019**, *6*, 1901328.
[26] H. Zou, B. He, P. Kuang, J. Yu, K. Fan, *ACS Appl. Mater. Interfaces* **2018**, *10*, 22311.
[27] X.-D. Zhu, Y. Xie, Y.-T. Liu, *J. Mater. Chem. A* **2018**, *6*, 21255.
[28] N. Li, Y. Zhang, M. Jia, X. Lv, X. Li, R. Li, X. Ding, Y.-Z. Zheng, X. Tao, *Electrochim. Acta* **2019**, *326*, 134976.
[29] Z. Liu, X. Liang, F. Ma, Y. Xiong, G. Zhang, G. Chen, L. Zhen, C. Xu, *Adv. Energy Mater.* **2023**, *13*, 2203609.
[30] D. A. Kuznetsov, Z. Chen, P. M. Abdala, O. V. Safonova, A. Fedorov, C. R. Müller, *J. Am. Chem. Soc.* **2021**, *143*, 5771.
[31] L. Jiang, J. Duan, J. Zhu, S. Chen, M. Antonietti, *ACS Nano* **2020**, *14*, 2436.
